# Gridded fossil CO_2_ emissions and related O_2_ combustion consistent with national inventories 1959–2018

**DOI:** 10.1038/s41597-020-00779-6

**Published:** 2021-01-07

**Authors:** Matthew W. Jones, Robbie M. Andrew, Glen P. Peters, Greet Janssens-Maenhout, Anthony J. De-Gol, Philippe Ciais, Prabir K. Patra, Frederic Chevallier, Corinne Le Quéré

**Affiliations:** 1 Tyndall Centre for Climate Change Research, School of Environmental Sciences, University of East Anglia, Norwich, NR4 7TJ UK; 2 CICERO Center for International Climate Research, Oslo, 0349 Norway; 3 European Commission, Joint Research Centre (JRC), Via E. Fermi 2749 (T.P. 123), 21027 Ispra, Varese Italy; 4 Laboratoire des Sciences du Climat et de l’Environnement, Institut Pierre-Simon Laplace, CEA-CNRS-UVSQ, CE Orme des Merisiers, 91191 Gif sur Yvette, France; 5 Research Institute for Global Change, Japan Agency for Marine-Earth Science and Technology (JAMSTEC), Yokohama, 236 0001 Japan

**Keywords:** Atmospheric chemistry, Climate and Earth system modelling, Carbon cycle, Atmospheric chemistry

## Abstract

Quantification of CO_2_ fluxes at the Earth’s surface is required to evaluate the causes and drivers of observed increases in atmospheric CO_2_ concentrations. Atmospheric inversion models disaggregate observed variations in atmospheric CO_2_ concentration to variability in CO_2_ emissions and sinks. They require prior constraints fossil CO_2_ emissions. Here we describe GCP-GridFED (version 2019.1), a gridded fossil emissions dataset that is consistent with the national CO_2_ emissions reported by the Global Carbon Project (GCP). GCP-GridFEDv2019.1 provides monthly fossil CO_2_ emissions estimates for the period 1959–2018 at a spatial resolution of 0.1°. Estimates are provided separately for oil, coal and natural gas, for mixed international bunker fuels, and for the calcination of limestone during cement production. GCP-GridFED also includes gridded estimates of O_2_ uptake based on oxidative ratios for oil, coal and natural gas. It will be updated annually and made available for atmospheric inversions contributing to GCP global carbon budget assessments, thus aligning the prior constraints on top-down fossil CO_2_ emissions with the bottom-up estimates compiled by the GCP.

## Abstract (summary)

**Table:** 

Measurement(s)	carbon dioxide emission • oxygen combustion
Technology Type(s)	digital curation • computational modeling technique
Factor Type(s)	annual and monthly fossil carbon dioxide emissions estimates • annual and monthly oxygen combustion estimates
Sample Characteristic - Environment	climate system
Sample Characteristic - Location	global

Machine-accessible metadata file describing the reported data: 10.6084/m9.figshare.13333643

## Background & Summary

Fossil fuel use, cement production and land-use change have perturbed the natural carbon cycle and increased the concentration of carbon dioxide (CO_2_) in the Earth’s atmosphere by almost 50% since 1750, from 277 ppm in 1750 to 407 ppm in 2018^1–3^. Routine assessment of the global carbon cycle is required to monitor the ongoing increases in atmospheric CO_2_ concentrations, evaluate the causes and drivers of this trend, and quantify the impact of policies that aim to stabilise and reverse it^3–5^. The global carbon budget (GCB) was evaluated on multi-year time scales by each of the foregoing Intergovernmental Panel on Climate Change (IPCC) assessment reports^6–10^, while the Global Carbon Project (GCP) has published an annual assessment of the GCB on an annual time-scale for over a decade^3,11–13^.

The GCP disaggregates the annual GCB into six components: atmospheric growth (G_ATM_); CO_2_ emissions due to fossil fuel combustion, non-combustion uses of fossil fuels, and cement production (E_FF_); CO_2_ emissions due to land-use change (E_LUC_); uptake of CO_2_ by the global ocean (S_OCEAN_); uptake of CO_2_ by the terrestrial biosphere (S_LAND_), the two later fluxes from ocean and land carbon models, respectively; and a budget imbalance term (B_IM_). G_ATM_ is the most precisely constrained term of the budget (1σ of 4%)^3^, while E_FF_, E_LUC_, S_OCEAN_ and S_LAND_ rely on analysis of national emissions reports^14–16^, satellite observations^17,18^, and process-based models^3,19,20^ and are more uncertain. If E_FF_, E_LUC_, S_OCEAN_ and S_LAND_ were perfectly constrained then their sum would be equal to the measured change in the atmospheric stock of CO_2_ (G_ATM_). However, the independent analysis of G_ATM_, E_FF_, E_LUC_, S_OCEAN_ and S_LAND_ using different methodologies results in an unconstrained budget, and a small budget imbalance term (B_IM_) is required to close the budget. The global carbon budget is thus closed as follows (S_OCEAN_ and S_LAND_ hold negative values)^3^:

1 $${G}_{ATM}={E}_{FF}+{E}_{LUC}+{S}_{OCEAN}+{S}_{LAND}+{B}_{IM}$$

Inversion models use an integrated approach to simultaneously quantify all fluxes of the global carbon budget and they are, by design, constrained by observations of atmospheric CO_2_ mole fraction, or satellite derived products of column CO_2_. Inversion models prescribe the fossil carbon emissions (E_FF_) because the current density of the surface network and the sampling of the atmosphere by satellites is too sparse to quantify this flux separately, and then estimate the total land flux (F_LAND_ = S_LAND_ + E_LUC_) and the ocean sink (S_OCEAN_) using a modelling framework that minimises data–model mismatch across all fluxes according to a cost function (see examples in refs. ^21–32^ and studies cited therein). By synchronously quantifying E_FF_, F_LAND_ and S_OCEAN_, inversion models avoid budget imbalance and hence the global carbon budget equation is closed without a B_IM_ term as follows:

2 $${G}_{ATM}={E}_{FF}^{inv}+{F}_{LAND}^{inv}+{S}_{OCEAN}^{inv}$$

Inversion models require prior constraints on the regional distribution of the CO_2_ fluxes that they seek to disaggregate. Here we describe our development of the Global Carbon Budget Gridded Fossil Emissions Dataset (GCP-GridFED; version 2019.1), a new gridded 0.1° × 0.1° global dataset of monthly CO_2_ emissions resulting from fossil fuel oxidation and the calcination of limestone during cement production. The gridded nation- and source- specific emissions in GCP-GridFED are consistent with the nation- and source- specific emissions inventories compiled for the GCP’s 2019 GCB assessment^3,33^ and for version 2019.1 cover the period 1959-2018. The GCP-GridFED will be updated each year for use by inversion models contributing to the annual updates of the GCB, thus aligning the prior constraints on top-down estimates of fossil CO_2_ emissions with the bottom-up estimates used by the GCP.

Gridded estimates of uncertainty in CO_2_ emissions are provided as an additional layer of GCP-GridFED and are based on the relative uncertainties (1σ) in fossil CO_2_ presented in the uncertainty assessment of the GCB^3^ and the relative uncertainties amongst emission sectors^34^. Uncertainties associated with the spatial disaggregation of national emissions are not included (see ‘CO_2_ Emissions Uncertainty’). Our approach to uncertainty quantification is broadly representative of the sectoral contributions to total emissions in each grid cell, which changes throughout the time series, and of differences in uncertainty across national emission reports. Inversion models may utilise these uncertainty grids but with the freedom to build more complex covariance structures to suit their requirements.

The global cycles of carbon and oxygen are coupled through their dual involvement in carboxylation reactions (photosynthesis), which consume CO_2_ and emit O_2_, and oxidation reactions (respiration and combustion), which consume O_2_ and emit CO_2_ (refs. ^35,36^). In addition to CO_2_ alone, some inversion models are able to constrain surface fluxes of O_2_ or atmospheric potential oxygen (APO ≈ O_2_ + 1.1CO_2_)^37–40^. Such models can utilise dual atmospheric measurements of CO_2_ and O_2_ and dual priors for CO_2_ and O_2_ surface fluxes and synchronously minimise data–model mismatch with respect to CO_2_ and O_2_. Alternatively, O_2_ fluxes can be constrained independently using atmospheric O_2_ observations and O_2_ surface flux priors. GCP-GridFED includes dual estimates of atmospheric O_2_ uptake due to the oxidation of fossil fuels, with the aim of supporting the inverse modelling of O_2_ or APO and with the view that the data can be used in multi-decadal analyses of the global oxygen budget. Our O_2_ uptake estimates are based on the oxidative ratios (OR; uptake of O_2_/emission of CO_2_)^36^ applied to the CO_2_ emission estimates for coal, oil, natural gas oxidation^36^.

## Methods

### Overview

GCP-GridFED was produced by scaling monthly gridded emissions for the year 2010, from the Emissions Database for Global Atmospheric Research (EDGAR; version 4.3.2)^41^, to the national annual emissions estimates compiled as part of the 2019 global carbon budget (GCB-NAE) for the years 1959–2018 (ref. ^3^). EDGAR data for the year 2010 is used because monthly gridded data was only available for this year at the time of product development (new data for 2015 was published recently and will be adopted in future versions of GCP-GridFED)^42^. We describe the key features of the EDGAR and GCB-NAE datasets below (see ‘Input Datasets’).

**Fig. 1:**
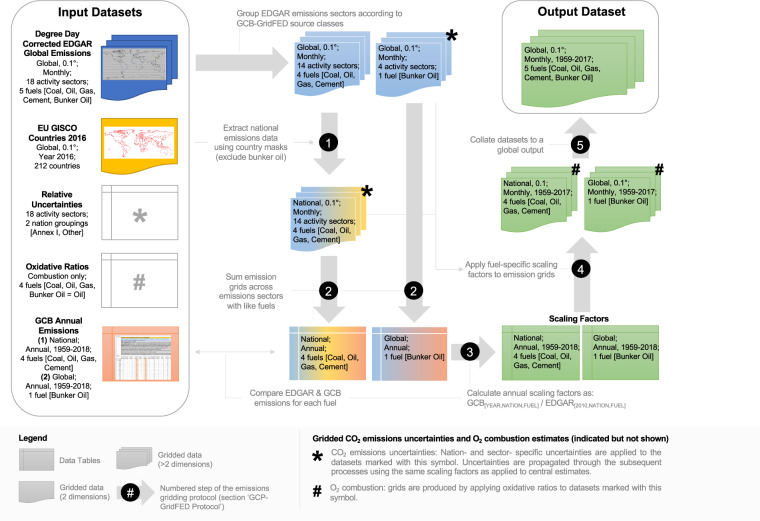
A conceptual depiction of the emissions scaling protocol used to produce GCP-GridFED as described in section 2.2. Descriptions of the input datasets are provided in section 2.1. This figure does not depict the procedure used to calculate emissions uncertainty or O_2_ combustion; however, the figure indicates the stages at which these additional outputs are produced within the CO_2_ emissions scaling protocol (marked as ‘*’ and ‘#’, respectively). Uncertainties in CO_2_ concentration are calculated for the EDGAR dataset in the year 2010 and scaled using the same factors as the central estimates for CO_2_ emission. O_2_ combustion estimates are calculated using oxidative ratios applied to the CO_2_ emissions from fuel combustion, prior to the final step of the protocol. Full details of the procedure used to produce gridded CO_2_ uncertainties and O_2_ combustion estimates can be found in sections 2.3 and 2.4, respectively.

GCB-NAE and EDGAR provide information regarding the global emission of CO_2_ through the combustion of fossil fuels, industrial processes and cement production, and some other minor sources (e.g. consumption of lubricants and paraffin waxes, solvent use, agricultural liming); nonetheless, their merits differ. GCB-NAE provides a consistent long-term dataset of annual national CO_2_ emissions (1750–2018), however this dataset is not spatially-explicit below the country level and does not include sub-annual variability in CO_2_ emissions. EDGAR provides estimates at high spatial resolution for specific fuels and sectors with a representation of the monthly distribution of emissions. However the EDGARv4.3.2 estimates are only available for 1970–2010 and a constant monthly distribution, matching the year 2010, is used throughout the time series^41^. Our approach merged these two complementary datasets to create a long-term (1959–2018) and gridded (0.1° × 0.1°) dataset of global monthly CO_2_ emissions. The start year of 1959 aligns with the period of direct atmospheric measurements of CO_2_ concentration^43^. Our approach is to scale EDGAR’s 2010 monthly gridded CO_2_ emissions to match the annual gridded CO_2_ emissions from GCB-NAE on a nation- and fuel- specific basis (see ‘emissions scaling protocol’, Fig. 1; Table 1).

**Table 1:** The relation of GCP-GridFED source classes to EDGAR activity sectors.

		GCP-GridFED Source Classes				
		Oil	Natural gas	Coal	Bunker Oil	Cement
**EDGAR Activity Sectors**	**Power Industry**	1A1a_OIL	1A1a_GAS	1A1a_COAL		
	**Fuel Transformation Industries**	1A1b_1A1c_1A5b1_1B1b_1B2a5_1B2a6_1B2b5_2C1b_OIL	1A1b_1A1c_1A5b1_1B1b_1B2a5_1B2a6_1B2b5_2C1b_GAS	1A1b_1A1c_1A5b1_1B1b_1B2a5_1B2a6_1B2b5_2C1b_COAL		
	**Manufacturing**	1A2_OIL	1A2_GAS	1A2_COAL		
	**Buildings**	1A4_OIL	1A4_GAS	1A4_COAL		
	**Transport: road**	1A3b_OIL				
	**Transport: Rail, Pipelines, Off-Road**	1A3c_1A3e (a)				
	**Fuel Exploitation**	1B1a_1B2a1_1B2a2_1B2a3_1B2a4_1B2c_OIL	1B1a_1B2a1_1B2a2_1B2a3_1B2a4_1B2c_GAS			
	**Production of Iron and Steel**			2C1a_2C1c_2C1d_2C1e_2C1f_2C2 (b)		
	**Production of Non-ferrous Metals**			2C3_2C4_2C5 (b)		
	**Fossil Fuel Fires**			7 A (c)		
	**Aviation**	1A3a_CRS_domestic			1A3a_CRS_international	
	**(Cruising)**					
	**Aviation (Landing and Take-Off)**	1A3a_LTO_domestic			1A3a_LTO_international	
	**Aviation (Climbing & Descent)**	1A3a_CDS_domestic			1A3a_CDS_international	
	**Shipping**				1A3d_1C2 (d)	
	**Non-energy Use of Fuels**	2 G (e)				
	**Solvents and Product Use**	3 (f)				
	**Chemical Processes**		2B (g)			
	**Non-metallic Minerals Production**					2 A (h)

The filename of the EDGAR grid layers is shown for each EDGAR activity sector. For a full description of each EDGAR sector, see Janssens-Maenhout *et al*. (ref. ^41^).

Assumptions made as to the fuels contributing to unstratified EDGAR sectors are as follows (see section 2.1.2 for further detail):

(a) all emissions from off-road, rail and pipeline transport relate to the combustion of oil.

(b) all emissions from the production of steel, iron and non-ferrous metals relate to the combustion of coal.

(c) all emissions from fossil fuel fires relate to underground coal fires.

(d) all emissions from shipping were assumed to relate to bunker oil combustion.

(e) all emissions from the non-energy use of fuels sector relate to non-combustion use of oil.

(f) all emissions of from the solvents and product use sector relate to non-combustion use of oil.

(g) all chemical process emissions relate to the non-combustion use of natural gas.

(h) all non-metallic minerals production from EDGAR are assumed to be emissions from cement (clinker) production.

GCP-GridFED includes additional data layers that are beneficial to inversion models. Gridded uncertainty in CO_2_ emissions from each nation and emissions sector is also propagated to our nation-, year- and fuel- specific emissions estimates (Table 2). Gridded estimates of the uptake of O_2_ related to oil, coal and natural gas use are also made using the literature-based oxidative ratios presented in the CO_2_ release and Oxygen uptake from Fossil Fuel Emission Estimate (COFFEE) dataset^36^.

**Table 2:** Calculation of uncertainties for each sector in GCP-GridFEDv2019.1.

TNO Sector	GCP-GridFED source classes included (a)	U_TNO: Relative Uncertainty Estimate	U_TNO: Sector-level Uncertainty ÷ Total Uncertainty	U_GridFED: Annex I Countries (b)	U_GridFED: Other Countries (c)
**Public power**	1A1a_OIL	86.4%	7.6	38%	76%
	1A1a_NATURAL GAS	86.4%	7.6	38%	76%
	1A1a_COAL	86.4%	7.6	38%	76%
**Industry**	1A2_OIL	18.2%	1.6	8%	16%
	1A2_NATURAL GAS	18.2%	1.6	8%	16%
	1A2_COAL	18.2%	1.6	8%	16%
	2C1a_2C1c_2C1d_2C1e_2C1f_2C2	18.2%	1.6	8%	16%
	2C3_2C4_2C5	18.2%	1.6	8%	16%
	2B	18.2%	1.6	8%	16%
	1A1b_1A1c_1A5b1_1B1b_1B2a5_1B2a6_1B2b5_2C1b_OIL	18.2%	1.6	8%	16%
	1A1b_1A1c_1A5b1_1B1b_1B2a5_1B2a6_1B2b5_2C1b_NATURAL GAS	18.2%	1.6	8%	16%
	1A1b_1A1c_1A5b1_1B1b_1B2a5_1B2a6_1B2b5_2C1b_COAL	18.2%	1.6	8%	16%
**Other stationary combustion**	1A4_OIL	15.4%	1.4	7%	14%
	1A4_NATURAL GAS	15.4%	1.4	7%	14%
	1A4_COAL	15.4%	1.4	7%	14%
**Fugitive**	1B1a_1B2a1_1B2a2_1B2a3_1B2a4_1B2c_OIL	34.1%	3.0	15%	30%
	1B1a_1B2a1_1B2a2_1B2a3_1B2a4_1B2c_NATURAL GAS	34.1%	3.0	15%	30%
	7 A	34.1%	3.0	15%	30%
**Solvents**	2 G	50%	4.4	22%	44%
	3	50%	4.4	22%	44%
**Road transport**	1A3b_OIL	13.9%	1.2	6%	12%
**Shipping**	1A3d_1C2	7.9%	0.7	3%	7%
**Aviation**	1A3a_CDS	9.8%	0.9	4%	9%
	1A3a_CRS	9.8%	0.9	4%	9%
	1A3a_LTO	9.8%	0.9	4%	9%
**Off-road transport**	1A3c_1A3e	22.4%	2.0	10%	20%
**Cement (not included in the TNO dataset)**	2 A			5%	10%
**Total Uncertainty**		11.3%		5%	10%

Calculations are based on (i) the ratio of the uncertainty in emissions from each sector (U_TNO_s_) to the uncertainty in total emissions (U_TNO_Tot_) and (ii) the relative uncertainty in emissions for Annex I countries (5%) and other countries (10%) from GCB-NAE^3^.

(a) GCP-GridFED source class codes are adopted from EDGAR. See Table 1 for more information.

(b) 5% uncertainty in total emissions.

(c) 10% uncertainty in total emissions.

We provide Figs. 2–12, the descriptive details in Tables 3 and 4, the summary statistics in Tables 5–7 and Online-Only Table 1 to outline the key features of GCP-GridFEDv2019.1 and assist with its technical validation.

**Fig. 2:**
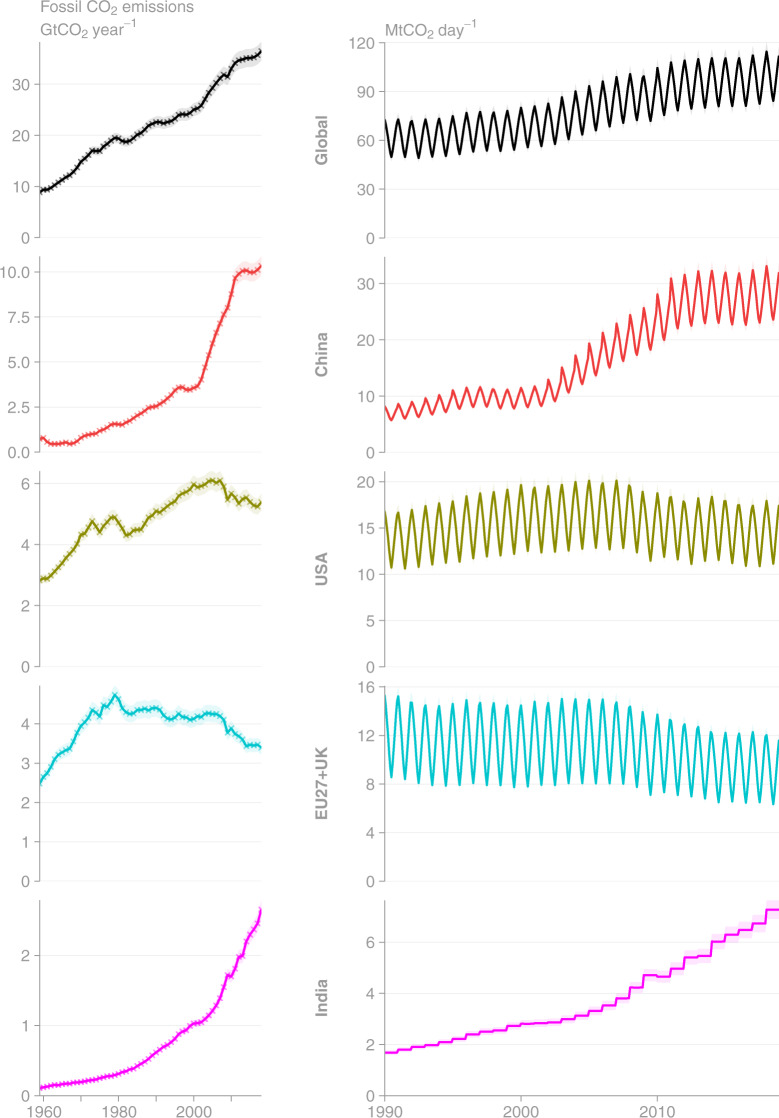
Time series of (left column) annual CO_2_ emissions (Gt CO_2_ year^−1^) and (right column) monthly fossil CO_2_ emissions (Mt CO_2_ day^−1^) as estimated by GCP-GridFED. Uncertainties in CO_2_ emissions are treated as 5% for Annex I nations and global, following the GCB uncertainty assessment^3^. (Top row) Total global emissions are disaggregated to (other rows) the top 4 emission regions. Crosses mark input data directly from GCB-NAE^3,33^.

**Fig. 3:**
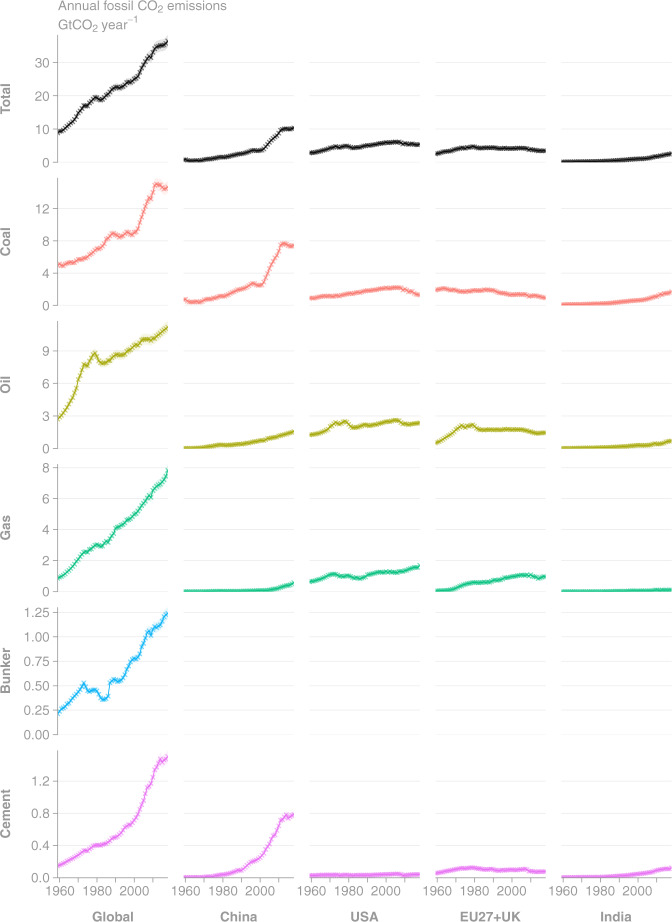
Time series of annual CO_2_ emissions as estimated by GCP-GridFEDv2019.1 for the period 1959–2018 (Gt CO_2_ year^−1^). Uncertainties in CO_2_ emissions are treated as 5% for Annex I nations and global, following the GCB uncertainty assessment^3^. (Top row, Left column) Total global emissions are disaggregated to the (other columns) top 4 emission regions and (other rows) source classes. Crosses mark input data directly from GCB-NAE^3,33^.

**Fig. 4:**
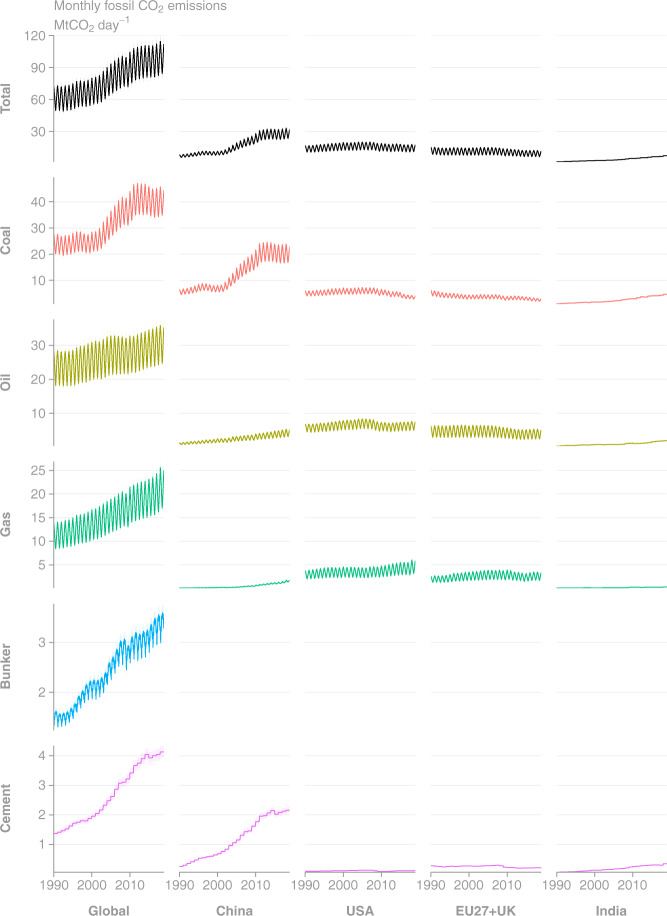
Time series of monthly CO_2_ emissions as estimated by GCP-GridFEDv2019.1. for the period 1990–2018 (Mt CO_2_ day^−1^). Uncertainties in CO_2_ emissions are treated as 5% for Annex I nations and global, following the GCB uncertainty assessment^3^. (Top row, Left column) Total global emissions are disaggregated to the (other columns) top 4 emission regions and (other rows) source classes.

**Fig. 5:**
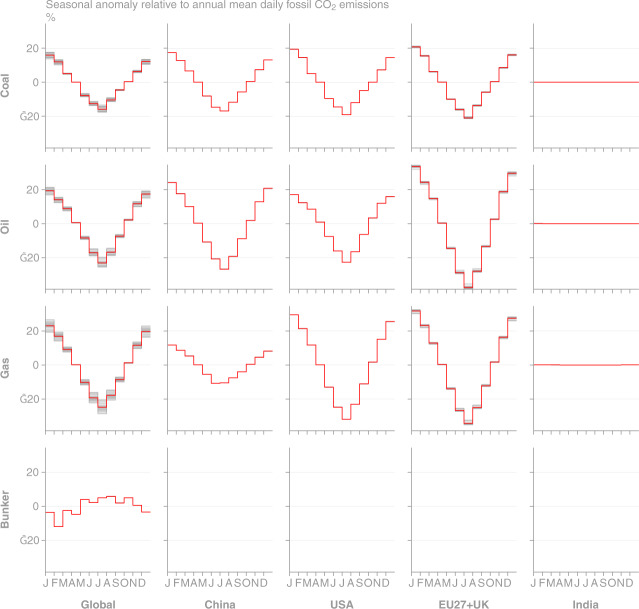
Monthly emissions anomaly relative to the annual mean daily emissions rate from GCP-GridFEDv2019.1. Each grey line represents a year of data in the period 1959–2018. The red line marks the mean value across the time series. (Top row, left column) Global seasonality is disaggregated to (other columns) the top 4 emission regions and (other rows) source classes. No seasonality is present in India, based on the EDGARv4.3.2 gridded input data for the year 2010.

**Fig. 6:**
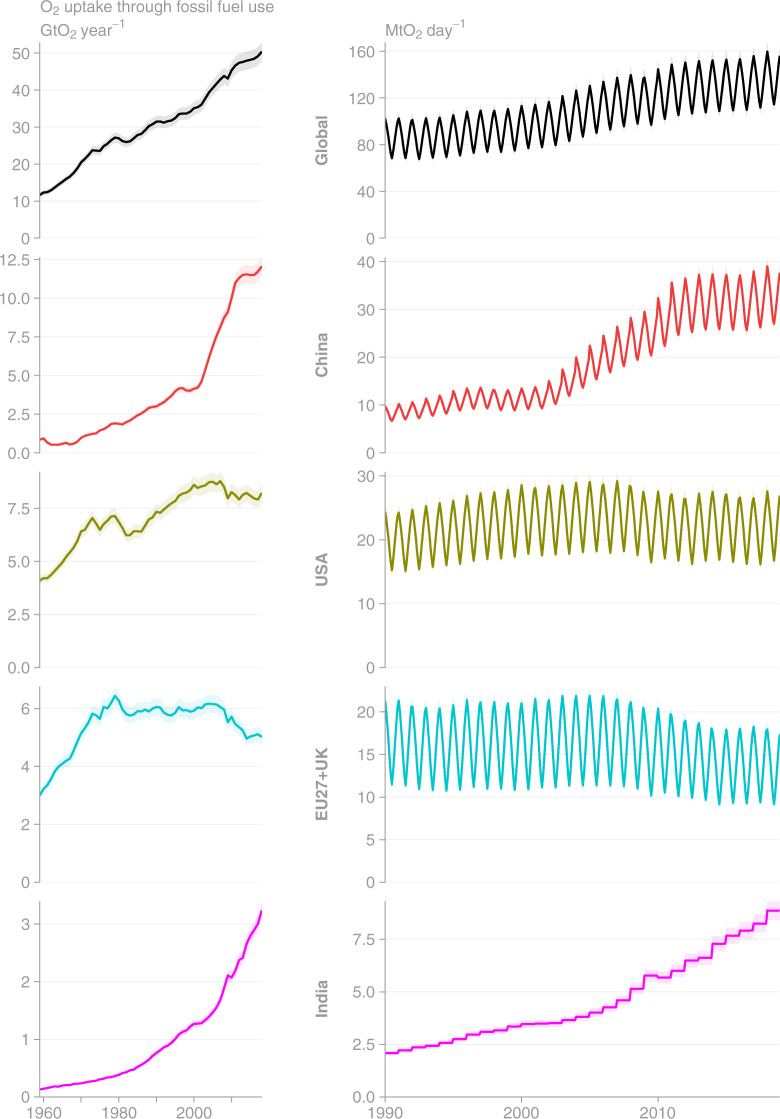
Time series of (left column) annual O_2_ uptake (Gt O_2_ year^−1^) and (right column) monthly O_2_ uptake (Mt O_2_ day^−1^) through fossil fuel oxidation as estimated by GCP-GridFED. (Top row) Total global uptake is disaggregated to (other rows) the top 4 emission regions. The plotted uptake uncertainties are based on CO_2_ emissions uncertainties of 5% for Annex I nations and global^3^, and they do not include uncertainties in oxidative ratios.

**Fig. 7:**
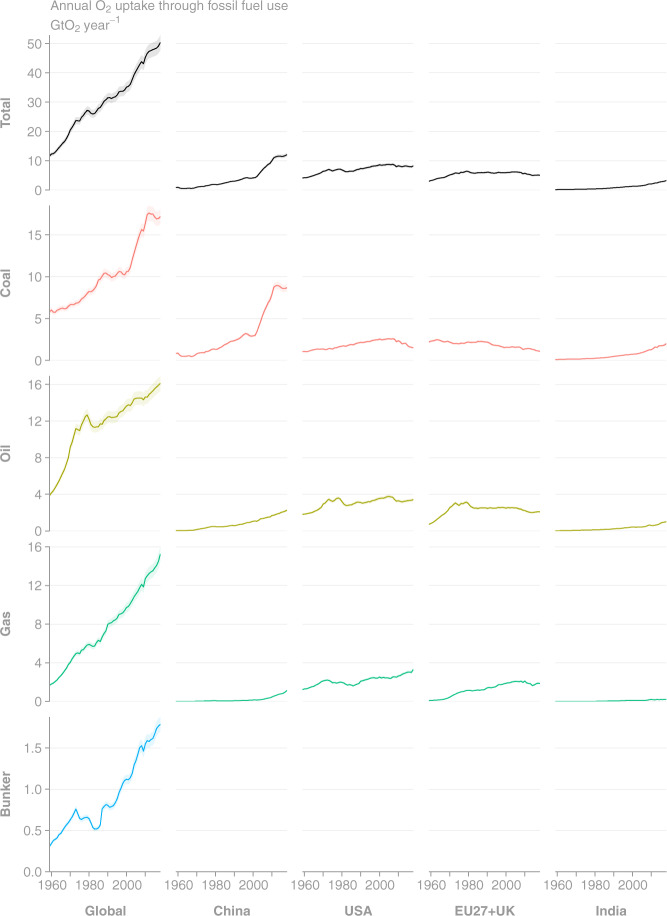
Time series of annual O_2_ uptake as estimated by GCP-GridFEDv2019.1 for the period 1959–2018 (Gt O_2_ year^−1^). The plotted uptake uncertainties are based on CO_2_ emissions uncertainties of 5% for Annex I nations and global^3^, and they do not include uncertainties in oxidative ratios. (Top row, Left column) Total global uptake is disaggregated to the (other columns) top 4 emission regions and (other rows) source classes.

**Fig. 8:**
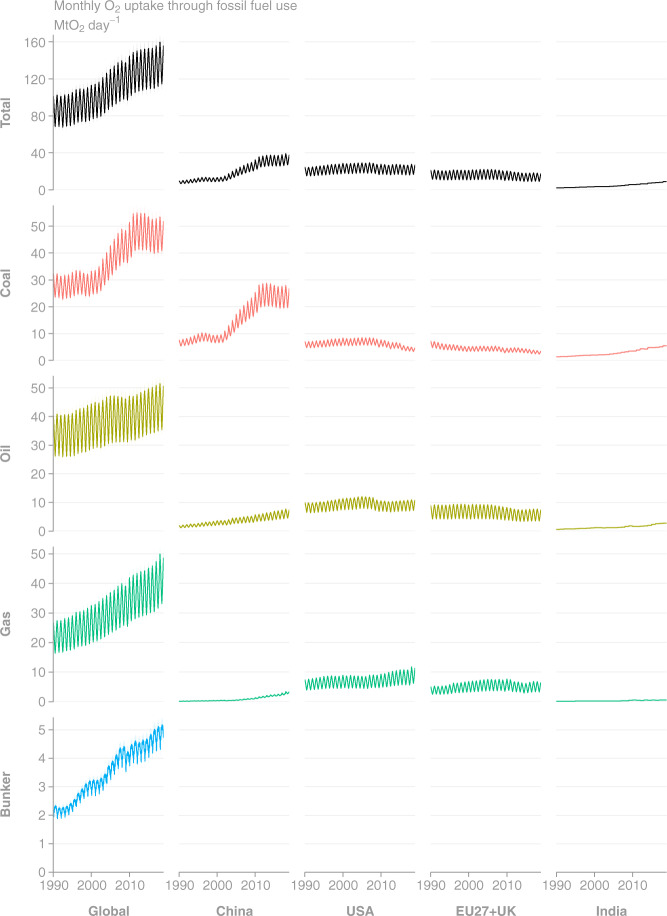
Time series of monthly O_2_ uptake as estimated by GCP-GridFEDv2019.1 for the period 1990–2018 (Mt O_2_ day^−1^). (Top row, Left column) Total global uptake is disaggregated to the (other columns) top 4 emission regions and (other rows) source classes. The plotted uptake uncertainties are based on CO_2_ emissions uncertainties of 5% for Annex I nations and global^3^, and they do not include uncertainties in oxidative ratios.

**Fig. 9:**
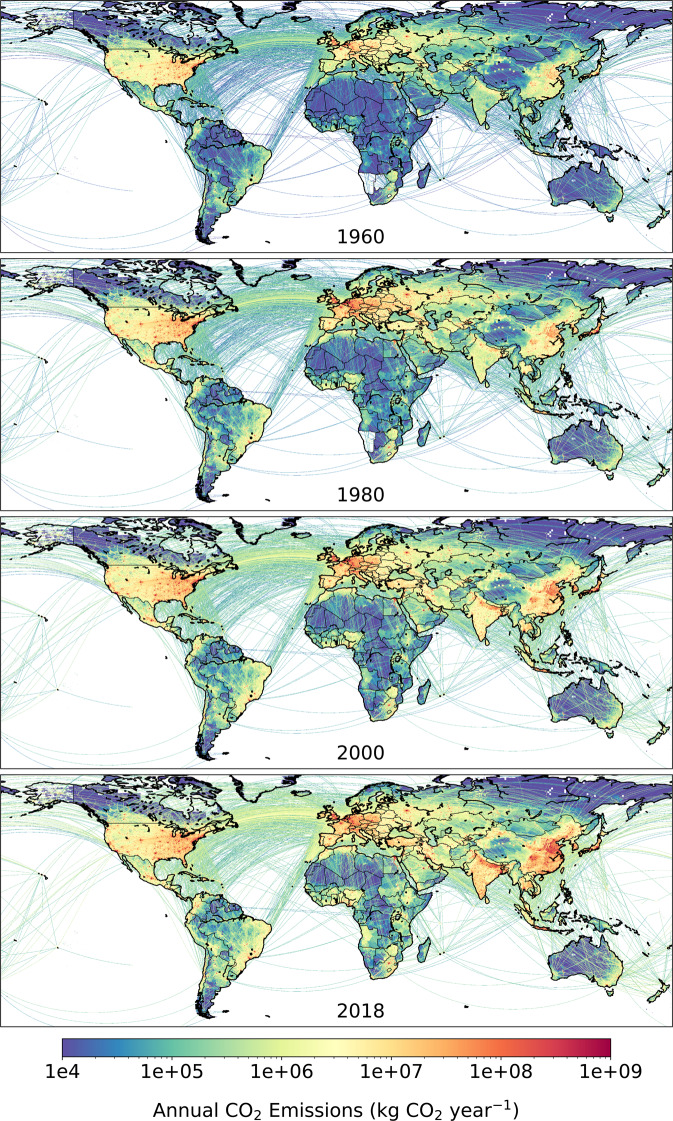
Spatial distribution of CO_2_ emissions as estimated by GCP-GridFED (kg CO_2_ year^−1^). Gridded (0.1° × 0.1°) estimates of total fossil CO_2_ emissions are shown for four years of the analytical period.

**Fig. 10:**
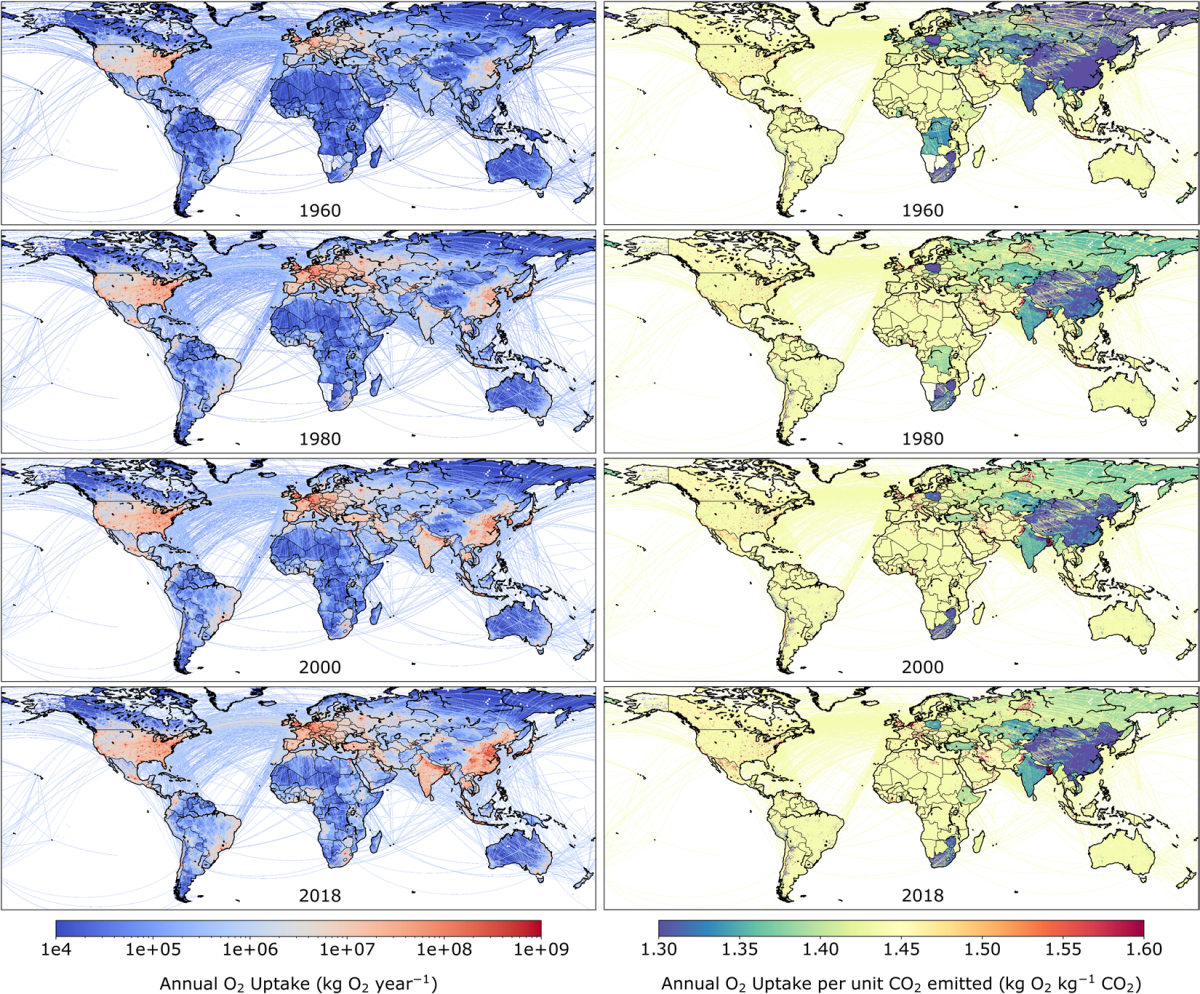
(Left panels) Spatial distribution of O_2_ uptake through fossil fuel use as estimated by GCP-GridFEDv2019.1 (kg O_2_ year^−1^). Gridded (0.1° × 0.1°) estimates of total O_2_ uptake are shown for four years of the time series (1959–2018). (Right panels) Spatially-explicit oxidative ratios (OR; kg O_2_ kg^−1^ CO_2_) for total emissions activities as estimated by GridFEDv2019.1.

**Fig. 11:**
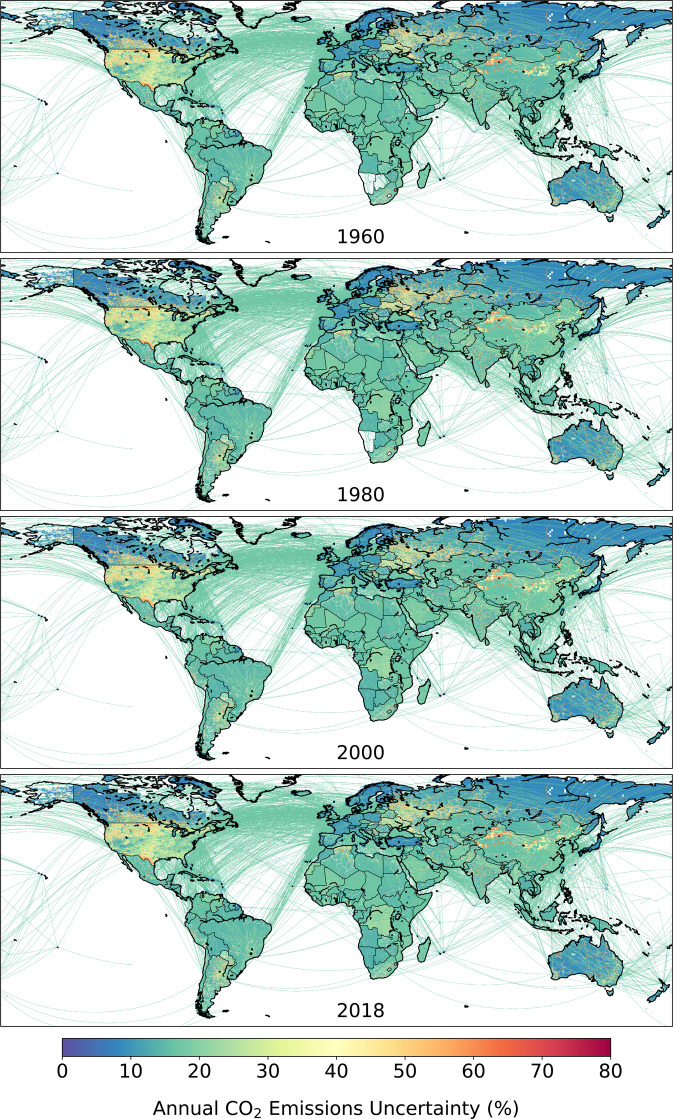
Gridded (0.1° × 0.1°) estimates of relative uncertainty in total CO_2_ emissions for four years of the GCP-GridFEDv2019.1 time series. Uncertainty in total emissions is aggregated from the sector-level estimates (see Table 2). The uncertainty estimates account for uncertainty across national emission reports and spatial differences in the sectoral breakdown to total emissions in each grid cell, which changes throughout the time series, however they exclude uncertainties associated with the spatial or temporal (monthly) disaggregation of national emissions (see ‘CO_2_ emissions uncertainty’). Aggregation of uncertainties to a coarser resolution should account for the non-independence of gridded emissions uncertainties.

**Fig. 12:**
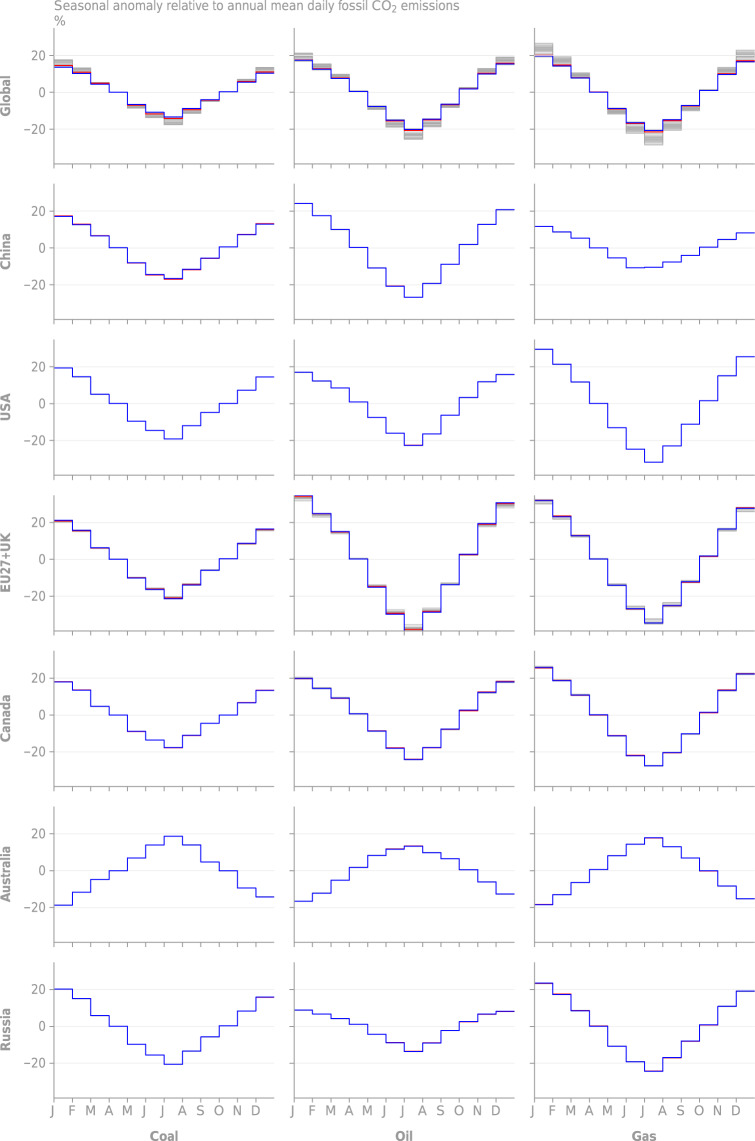
Monthly emissions anomaly relative to the annual mean daily emissions rate from GCP-GridFEDv2019.1. Each grey line represents a year of data in the period 1959–2018. The red line marks the marks 2010 and is compared with values obtained directly from the EDGARv4.3.2 grids for the year 2010. (Top row, left column) Global seasonality is disaggregated to (other columns) source classes (other rows) regions.

**Table 3:** Table of groups, variables, dimensions and units of the output files with naming convention GCP_Global_{YYYY}.nc. Numbers show the length of each dimensions for each variable.

				Dimensions		
				Time	Latitude	Longitude
Groups	Variables	Description	Unit	month of the year, days since the first day of YYYY	Degrees North of the equator (cell centres)	Degrees East of the Prime Meridian (cell centres)
CO_2_	COAL	Monthly emissions of CO_2_ in each cell (kg CO_2_ month-1) from COAL	kg CO_2_ month-1	12	1,800	3,600
	OIL	Monthly emissions of CO_2_ in each cell (kg CO_2_ month-1) from OIL	kg CO_2_ month-1	12	1,800	3,600
	GAS	Monthly emissions of CO_2_ in each cell (kg CO_2_ month-1) from GAS	kg CO_2_ month-1	12	1,800	3,600
	CEMENT	Monthly emissions of CO_2_ in each cell (kg CO_2_ month-1) from CEMENT	kg CO_2_ month-1	12	1,800	3,600
	BUNKER	Monthly emissions of CO_2_ in each cell (kg CO_2_ month-1) from BUNKER	kg CO_2_ month-1	12	1,800	3,600
CO_2__uncertainty	COAL	Uncertainty in monthly emissions of CO_2_ in each cell (kg CO_2_ month-1) from COAL	kg CO_2_ month-1	12	1,800	3,600
	OIL	Uncertainty in monthly emissions of CO_2_ in each cell (kg CO_2_ month-1) from OIL	kg CO_2_ month-1	12	1,800	3,600
	GAS	Uncertainty in monthly emissions of CO_2_ in each cell (kg CO_2_ month-1) from GAS	kg CO_2_ month-1	12	1,800	3,600
	CEMENT	Uncertainty in monthly emissions of CO_2_ in each cell (kg CO_2_ month-1) from CEMENT	kg CO_2_ month-1	12	1,800	3,600
	BUNKER	Uncertainty in monthly emissions of CO_2_ in each cell (kg CO_2_ month-1) from BUNKER	kg CO_2_ month-1	12	1,800	3,600
O_2_	COAL	Monthly uptake of O_2_ in each cell (kg O_2_ month-1) due to COAL use	kg O_2_ month-1	12	1,800	3,600
	OIL	Monthly uptake of O_2_ in each cell (kg O_2_ month-1) due to OIL use	kg O_2_ month-1	12	1,800	3,600
	GAS	Monthly uptake of O_2_ in each cell (kg O_2_ month-1) due to GAS use	kg O_2_ month-1	12	1,800	3,600
	BUNKER	Monthly uptake of O_2_ in each cell (kg O_2_ month-1) due to BUNKER use	kg O_2_ month-1	12	1,800	3,600

**Table 4:** Table of groups, variables, dimensions and units of the output file GCP_Global_Annual.nc. Numbers show the length of each dimensions for each variable.

				Dimensions		
				Time	Latitude	Longitude
Groups	Variables	Description	Unit	days since 1959–01–01	Degrees North of the equator (cell centres)	Degrees East of the Prime Meridian (cell centres)
CO_2_	COAL	Annual emissions of CO_2_ in each cell (kg CO_2_ year-1) from COAL	kg CO_2_ year-1	60	1,800	3,600
	OIL	Annual emissions of CO_2_ in each cell (kg CO_2_ year-1) from OIL	kg CO_2_ year-1	60	1,800	3,600
	GAS	Annual emissions of CO_2_ in each cell (kg CO_2_ year-1) from GAS	kg CO_2_ year-1	60	1,800	3,600
	CEMENT	Annual emissions of CO_2_ in each cell (kg CO_2_ year-1) from CEMENT	kg CO_2_ year-1	60	1,800	3,600
	BUNKER	Annual emissions of CO_2_ in each cell (kg CO_2_ year-1) from BUNKER	kg CO_2_ year-1	60	1,800	3,600
CO_2__uncertainty	COAL	Uncertainty in Annual emissions of CO_2_ in each cell (kg CO_2_ year-1) from COAL	kg CO_2_ year-1	60	1,800	3,600
	OIL	Uncertainty in Annual emissions of CO_2_ in each cell (kg CO_2_ year-1) from OIL	kg CO_2_ year-1	60	1,800	3,600
	GAS	Uncertainty in Annual emissions of CO_2_ in each cell (kg CO_2_ year-1) from GAS	kg CO_2_ year-1	60	1,800	3,600
	CEMENT	Uncertainty in Annual emissions of CO_2_ in each cell (kg CO_2_ year-1) from CEMENT	kg CO_2_ year-1	60	1,800	3,600
	BUNKER	Uncertainty in Annual emissions of CO_2_ in each cell (kg CO_2_ year-1) from BUNKER	kg CO_2_ year-1	60	1,800	3,600
O_2_	COAL	Annual uptake of O_2_ in each cell (kg O_2_ year-1) due to COAL use	kg O_2_ year-1	60	1,800	3,600
	OIL	Annual uptake of O_2_ in each cell (kg O_2_ year-1) due to OIL use	kg O_2_ year-1	60	1,800	3,600
	GAS	Annual uptake of O_2_ in each cell (kg O_2_ year-1) due to GAS use	kg O_2_ year-1	60	1,800	3,600
	BUNKER	Annual uptake of O_2_ in each cell (kg O_2_ year-1) due to BUNKER use	kg O_2_ year-1	60	1,800	3,600
O_2__uncertainty	COAL	Annual uptake of O_2_ in each cell (kg O_2_ year-1) due to COAL use	kg O_2_ year-1	60	1,800	3,600
	OIL	Annual uptake of O_2_ in each cell (kg O_2_ year-1) due to OIL use	kg O_2_ year-1	60	1,800	3,600
	GAS	Annual uptake of O_2_ in each cell (kg O_2_ year-1) due to GAS use	kg O_2_ year-1	60	1,800	3,600
	BUNKER	Annual uptake of O_2_ in each cell (kg O_2_ year-1) due to BUNKER use	kg O_2_ year-1	60	1,800	3,600

**Table 5:** Regional summary statistics relating to total annual CO_2_ emissions from GridFEDv2019.1.

Variable	Period	Global	Northern Extratropics	Tropics	Southern Extratropics
Mean Annual Emissions (Gt CO_2_ year^−1^)	1960–1969	11.1	9.6	1.3	0.2
	1970–1979	17.2	14.4	2.5	0.3
	1980–1989	20.1	16.2	3.5	0.3
	1990–1999	23.2	17.7	5.1	0.4
	2000–2009	28.5	20.4	7.5	0.5
	2010–2018	34.9	23.3	10.9	0.6
	2018	36.4	23.8	11.9	0.6
Mean Fraction of Global Emissions (%)	1960–1969		86.1	12.1	1.5
	1970–1979		83.9	14.3	1.6
	1980–1989		80.7	17.3	1.7
	1990–1999		76.1	21.8	1.8
	2000–2009		71.5	26.3	1.9
	2010–2018		66.7	31.2	1.7
	2018		65.2	32.8	1.7
Mean Annual Emissions Uncertainty (Gt CO_2_ year^−1^)	1960–1969	2.7	2.2	0.4	0.0
	1970–1979	4.0	3.1	0.8	0.1
	1980–1989	5.1	3.8	1.2	0.1
	1990–1999	6.1	4.2	1.8	0.1
	2000–2009	7.9	5.0	2.7	0.1
	2010–2018	10.3	6.1	4.1	0.2
	2018	10.8	6.1	4.5	0.2
Mean Annual Emissions Uncertainty (%)	1960–1969	24.6	23.5	32.4	26.2
	1970–1979	23.2	21.6	32.7	24.8
	1980–1989	25.4	23.3	35.3	25.6
	1990–1999	26.3	23.7	35.7	25.7
	2000–2009	27.8	24.7	36.5	25.5
	2010–2018	29.5	26.0	37.2	25.2
	2018	29.6	25.8	37.5	24.9
Mean Annual Emissions Growth (%)	1960–1969	4.5	4.4	5.0	5.0
	1970–1979	3.6	3.3	5.7	3.5
	1980–1989	1.3	0.9	3.2	2.6
	1990–1999	0.9	0.2	3.5	2.3
	2000–2009	2.6	1.8	4.7	2.0
	2010–2018	1.7	1.1	2.9	0.6
	2018	2.1	1.7	3.1	−0.2

**Table 6:** Regional summary statistics relating to seasonality of total CO_2_ emissions from GridFEDv2019.1.

Variable	Period	Global	Northern Extratropics	Tropics	Southern Extratropics
Mean Daily Emissions (Mt CO_2_ day^−1^)	1960–1969	30.5	26.2	3.7	0.5
	1970–1979	47.1	39.5	6.7	0.7
	1980–1989	55.1	44.5	9.5	0.9
	1990–1999	63.6	48.4	13.9	1.2
	2000–2009	78.1	55.8	20.5	1.5
	2010–2018	95.6	63.8	29.8	1.7
	2018	99.8	65.1	32.7	1.7
Mean Seasonal Amplitude (Mt CO_2_ day^−1^)	1960–1969	6.3	6.1	0.1	0.3
	1970–1979	10.1	9.8	0.2	0.5
	1980–1989	11.0	10.7	0.2	0.5
	1990–1999	12.2	11.7	0.2	0.8
	2000–2009	13.9	13.1	0.3	1.4
	2010–2018	15.5	14.0	0.4	1.9
Mean Seasonal Amplitude (%)	1960–1969	20.9	23.6	20.7	7.7
	1970–1979	21.6	25.0	21.2	7.6
	1980–1989	20.0	24.1	21.0	5.6
	1990–1999	19.1	24.2	20.9	6.1
	2000–2009	18.0	23.4	21.0	6.8
	2010–2018	16.3	22.1	21.6	6.5

**Table 7:** Regional summary statistics relating to annual CO_2_ emissions from each source, from GridFEDv2019.1.

Variable	Period	Global	Northern Extratropics	Tropics	Southern Extratropics
Mean Daily Emissions (Mt CO_2_ day^−1^)	1960–1969	30.5	26.2	3.7	0.5
	1970–1979	47.1	39.5	6.7	0.7
	1980–1989	55.1	44.5	9.5	0.9
	1990–1999	63.6	48.4	13.9	1.2
	2000–2009	78.1	55.8	20.5	1.5
	2010–2018	95.6	63.8	29.8	1.7
	2018	99.8	65.1	32.7	1.7
Mean Seasonal Amplitude (Mt CO_2_ day^−1^)	1960–1969	6.3	6.1	0.1	0.3
	1970–1979	10.1	9.8	0.2	0.5
	1980–1989	11.0	10.7	0.2	0.5
	1990–1999	12.2	11.7	0.2	0.8
	2000–2009	13.9	13.1	0.3	1.4
	2010–2018	15.5	14.0	0.4	1.9
Mean Seasonal Amplitude (%)	1960–1969	20.9	23.6	20.7	7.7
	1970–1979	21.6	25.0	21.2	7.6
	1980–1989	20.0	24.1	21.0	5.6
	1990–1999	19.1	24.2	20.9	6.1
	2000–2009	18.0	23.4	21.0	6.8
	2010–2018	16.3	22.1	21.6	6.5

### Input datasets

#### National annual emissions from the global carbon budget 2019 (GCB-NAE)

The GCB estimates national annual emissions of CO_2_ due to coal, oil and natural gas combustion, the oxidative use of these fuels in non-combustive industrial processes, and the production of cement clinker^3,14–17,44^. National CO_2_ emissions are preferentially taken from the country submissions to the United Nations Framework Convention on Climate Change (UNFCCC) for 42 “Annex I” countries over the period 1990–2018^44^. These countries were members of the Organisation for Economic Co-operation and Development (OECD) in 1992, plus 16 non-OECD European countries and Russia, and contributed ~60% of total global emissions in 1990. Emissions in other countries and in Annex I countries prior to 1990 derive from the Carbon Dioxide Information Analysis Center (CDIAC)^15^ and are rooted in energy statistics published by the United Nations (UN)^16,45^. For recent years not covered by either the UNFCCC or CDIAC datasets, the national emissions are predicted using national or regional energy growth rates from the annual BP Statistical Review of World Energy^14^. National cement emissions are based on national inventories of cement production and ratios of clinker production from officially reported clinker production data and emission factors, IPCC default emission factors, industry-reported clinker production, and survey-based clinker ratios^16^.

#### Gridded monthly emissions from EDGAR

The Emissions Database for Global Atmospheric Research (EDGAR) version 4.3.2^41^ is a dataset of global emissions of gases and particulates, including CO_2_, based on available national statistics, default emission factors and methods recommended by IPCC^46,47^. EDGAR uses a bottom-up approach that calculates gridded (0.1° × 0.1°) monthly CO_2_ emissions for activity sectors based on: statistics that track national levels of each activity; proxy data representing the spatial and temporal distribution of each activity; the mix of technologies used to perform each activity; the fuel mix used by each technology, and; emissions factors for the technology and fuel combinations, which are also corrected for the emission control technologies in place. A detailed description of EDGAR’s gridding procedure is available elsewhere (refs. ^41,48^) however we summarise below the key features of its design:

- 28 EDGAR activity sectors are based on the 48 sectors defined by IPCC guidelines^46,47^.
- Activity in each sector is tracked from 1970–2015 using statistics that represent demand and supply of goods and energy, including: fuel-specific energy balances, fuel production, commodity production and cement clinker production and agriculture-related activities.
- Emission factors are taken from the guidelines issued by the IPCC^46,47^ and are assigned to each country in the following order of preference: national, regional, country group (Annex I/non-Annex I).
- National emissions of CO_2_ from each sector are distributed across months using sector-specific or, preferentially, technology-specific monthly shares.
- Emissions are distributed in space using spatial proxy data (that vary stepwise over time 1990–2010), such as population density, point source locations and transport routes.

Some of the uncertainties associated with using proxy data to disaggregate emission in time and space are considered in later sections (see ‘CO_2_ emissions uncertainty’).

### EDGAR sectors included in GCP-GridFED

Of the 28 EDGAR sectors, the 18 relating to fossil fuel combustion, non-combustion use of fossil fuel and cement production were used in GCP-GridFED. These 18 sectors were selected to correspond as closely as possible with the activities included in the GCB-NAE emission estimates. The 18 activity sectors incorporated from EDGAR into GCP-GridFED are shown in Table 1.

Where possible, emissions from each EDGAR sector were further separated into specific fuels using fuel-specific data from an intermediate processing step of the EDGAR gridding protocol^41^. Where this was not possible, it was necessary to make the assumptions that follow about the fuels that contribute to emissions in each sector. These assumptions are based on the sector descriptions provided in the IPCC guidelines^46,47^ and the major contributing activities and fuel dependencies in each sector. Specifically, we assume that:

- All chemical process emissions relate to the non-combustion use of natural gas.
- All emissions from the non-energy use of fuels sector relate to non-combustion use of oil. This sector chiefly comprises the use of waxes and lubricants.
- All emissions from the solvents and product use sector relate to non-combustion use of oil. This sector chiefly comprises ﻿solvents in paint, degreasing and dry cleaning, chemical products and other product use.
- All emissions from the production of steel, iron and non-ferrous metals relate to the oxidation of coal and production of cokes.
- All emissions from fossil fuel fires relate to underground coal fires. This sector also includes oil flaring emissions in Kuwait, however fossil fuel fire emissions were found to be negligible in Kuwait.
- All emissions from off-road, rail and pipeline transport relate to the combustion of oil.
- All emissions from the production of non-metallic minerals relate to cement clinker production.

National CO_2_ emissions data were extracted from the EDGAR datasets for the purpose of national annual emissions scaling. National masks were based on the ‘countries 2016’ dataset of the Geographic Information System of the European Commission (EU-GISCO)^49^.

The appropriate positioning of power plants is key to distributing total emissions accurately because the power sector accounts for ~45% of global emissions^50^. Changes in the available datasets of power plant geolocations are common, and hence we note the importance of recording which datasets are used in each release of gridded emissions products. GridFEDv2019.1 adopts point source geolocations from EDGAR v4.3.2, which are scaled as described below (see ‘GCP-GridFED Protocol’). The EDGAR protocol for geolocating power plant emissions is summarised as follows, with full documentation provided by Janssens-Maenhaut *et al*.^41^. The location, fuel type and seasonality of power plant emissions derives from the CARMAv3.0 dataset^51^. The 2010 gridded emissions dataset used here as the scaling basis includes over 60,000 plants mapped globally in CARMAv3.0 in the year 2007. Standard QA/QC screening was applied to the CARMAv3.0 dataset, including gap-filling of missing (0, 0) plant coordinates, correcting inverted (lon, lat) coordinates and adding some additional points for Russia. National power sector emissions for each fuel type are distributed across plants in proportion to their reported capacities. For larger countries (e.g. USA) with a non-uniform distribution of coal power plants, the fuel-specific distribution of emissions is considered a significant improvement over foregoing approaches. Emissions from each power plant reflect the fuel mix of the plant and the respective carbon intensity of emissions from that fuel mix. However, details of the technologies used by each plant, including carbon capture and storage, are not available. Alternative mappings of point sources can be based on night light detections by satellite^52^ or population data^53^ but these are least aligned with EDGAR’s ‘bottom up’ approach^41^.

#### Heating and cooling degree day (HCDD) Correction

The monthly distribution (seasonality) of global CO_2_ emissions is principally determined by seasonality of climate in the Northern Hemisphere, and thus a peak in emissions occurs in the boreal winter months and a trough occurs in the boreal summer months. Although this seasonality is predictable, inter-annual variability in weather influences the distribution of emission across the months. Because the monthly emissions distribution in the EDGAR dataset is derived only from 2010 data, we applied a correction to the EDGAR data to account for the impacts of inter-annual variability on emissions. Specifically, we used a heating and cooling degree day (HCDD) correction to implement inter-annual variability in the monthly distribution of CO_2_ emissions from selected EDGAR sectors (power industry, 1A1a; buildings, 1A4; manufacturing, 1A2; and road transport, 1A3b; see Table 1). The HCDD correction approach was implemented as follows.

First, monthly (*m*) HCDDs were calculated based on gridded (0.5° × 0.5°) daily mean temperature (*T*) data for the years 1959–2018 from the Climatic Research Unit time-series version 4.03 (CRU-TSv4.03)^54^ and following Spinoni *et al*. (refs. ^55,56^). For each 0.5° × 0.5° cell (*i_r*, *j_r*) of CRU-TSv4.03, HCDD was calculated as the absolute difference between the daily mean temperature of each month and an upper temperature threshold of 22 °C or a lower temperature threshold of 15.5 °C degrees^55,56^, multiplied by the number of days in the month (*d*).

3 $$HCD{D}_{m,i\_r,j\_r}=\left\{\begin{array}{c}\left(15.5-{\bar{T}}_{m,i\_r,j\_r}\right)\cdot d,{\bar{T}}_{m,i\_r,j\_r} < 15.5\\ \left.{\bar{T}}_{m,i\_r,j\_r}-22.0\right)\cdot d,{\bar{T}}_{m,i\_r,j\_r} > 22.0\end{array}\right.$$

Second, the monthly fraction of annual HCDDs (*HCDDfrac*) was calculated for each month in the year 2010 as follows.

4 $$HCDDfra{c}_{m,i\_r,j\_r}=\frac{HCD{D}_{m,i\_r,j\_r}}{\sum HCD{D}_{i\_r,j\_r}}$$

Third, the monthly fraction of annual emissions (*Efrac*) for each of the relevant sectors (*s*; 1A1a, 1A4, 1A2, 1A3b) was calculated on a reduced-resolution grid (0.5° × 0.5°; *i_r*, *j_r*) for each month as follows.

5 $$Efra{c}_{s,m,i\_r,j\_r}=\frac{EDGA{R}_{s,m,i\_r,j\_r}}{\sum EDGA{R}_{s,i\_r,j\_r}}$$

Fourth, a simple linear regression equation of the form below was fitted between monthly *HCDDfrac* and *Efrac* in the year 2010.

6 $$Efra{c}_{s,m,i\_r,j\_r}={a}_{s,i\_r,j\_r}+({b}_{s,i\_r,j\_r}\cdot HCDDfra{c}_{m,i\_r,j\_r})+erro{r}_{s,i\_r,j\_r}$$

The HCDD correction was implemented in each year of the time series by predicting *Efrac* based on *HCDDfrac*. The correction was applied only to cells where the R^2^ value of the linear regression equation exceeded 0.66 (where 66% of variation in *Efrac* was explained by variation in *HCDDfrac*). The following conditional approach was applied in all years (1959–2018).

7 $$EDGA{R}_{s,m,i,j}=\left\{\begin{array}{c}{a}_{s,i,j}+({b}_{s,i,j}\cdot HCDDfra{c}_{m,i,j}),{R}_{s,i\_r,j\_r}^{2}\ge 0.66\\ EDGA{R}_{s,m,i,j},else\end{array}\right.$$

Here *HCDDfrac*, *a* and *b* were re-gridded by repeating each grid cell in the *i_r*, *j_r* dimensions to provide output at the resolution of the EDGAR grid (0.1° × 0.1°; *i*, *j*).

### GCP-GridFED Protocol

#### CO_2_ Emissions

GCP-GridFED was generated using the six-step emissions scaling protocol set out below and applied sequentially for each year in the period 1959–2018 (see Fig. 1):

1.     **Group emissions from EDGAR sectors by source class.** The global gridded (*i*, *j*) monthly (*m*) CO_2_ emissions were summed across the EDGAR activity sectors (*s*) in each source class used in this study (*S*; see Table 1). The monthly distribution of annual emissions was adjusted in advance using Eq. 7.

8 $$EDGA{R}_{S,m,i,j}=\sum EDGA{R}_{s,m,i,j}$$

2.     **Extract gridded emissions data from EDGAR for each country.** A subset of gridded monthly CO_2_ emissions from each GCP-GridFED source class (see Table 1) was extracted for each country (*c*) using country masks (True/False) from the EU-GISCO dataset^49^. No subset was extracted for the bunker fuels source class; the entire grid layer was scaled globally. Hence, all grid cells were included in an ‘international’ mask (True in all cells) and treated thereafter in the same way as each country.

9 $$EDGA{R}_{S,m,i\left[c\right],j[c]}=EDGA{R}_{S,m,i\left[True\right],j[True]}$$

3.      **Sum EDGAR emissions for each GCP-GridFED source class.** Monthly CO_2_ emissions were summed both across the months of the year and across the grid extracted for each nation, for each GCP-GridFED source class. The resulting annual emission sub-totals were stored in a tabular format matching the structure of the GCB-NAE data.

10 $$EDGA{R}_{S,c}=\sum \sum {EDGAR}_{S,m,i\left[c\right],j[c]}$$

4.     **Calculate scaling factors based on comparison of EDGAR and GCB-NAE emissions estimates**. For each country *c* and for each source class *S*, the scaling factor (α) required to convert the annual CO_2_ emissions from EDGAR (step 3) to the annual CO_2_ emissions estimate from GCB-NAE was derived as follows.

11 $$GC{B}_{S,c}={\alpha }_{S,c}\cdot EDGA{R}_{S,c}$$

5.     **Apply annual scaling factors to monthly emission grids**. The scaling factors for each nation and GCP-GridFED source class were applied to the national monthly CO_2_ emissions grids generated in step 2. The same scaling factor was used for all months. For the bunker fuels source class, the scaling factor was applied to the equivalent global data.

12 $$GridFE{D}_{S,m,i[c],j[c]}={\alpha }_{S,c}\cdot EDGA{R}_{S,m,i[c],j[c]}$$

6.     **Collate national data to a global output**. Scaled monthly CO_2_ emissions grids from all nations were merged into a single grid for each GCP-GridFED source class.

We do not attempt to adjust the EDGARv4.3.2 grids (year 2010) for a range of historical changes to the spatial distribution of emissions, for instance due to the expansion of road networks or flight routes, the commissioning/decommissioning of facilities or large-scale population migration. The resolution of these issues will be prioritised in future developments to the GCP-GridFED protocol. We note that developments introduced in EDGARv5.0 (ref. ^42^) include refined spatial proxy records and national temporal profiles covering the period 1970–2012, which will support further developments to the GCP-GridFED protocol. Dedicated datasets of fuel-specific monthly CO_2_ emissions are also emerging for some countries, including India^57^ and the USA^58^, and could be used preferentially in the GCP-GridFED protocol. Additional sources such as the diffusive coal mine oxidation CO_2_, as derived for the dataset CHE-EDGARv4.3.2_FT2015^41,59,60^ will also be considered.

We do not consider emissions of non-CO_2_ carbon emissions that later influence atmospheric CO_2_ (in particular, CO and CH_4_). Here all fossil carbon is assumed to be emitted as fossil CO_2_, whereas a fraction is in reality emitted as CO and later represents a diffuse fossil CO2 source after oxidation to CO_2_ (~1.8 Pg CO_2_-equivalent year^−1^)^61^. In GridFEDv2019.1, the diffuse nature of this CO_2_ source is not considered, and the source is instead placed at the surface at the time and location of oxidation. Meanwhile, fossil CH_4_ fugitive emissions represent an additional diffuse source of CO_2_ emissions (0.4 Pg CO_2_-equivalent year^−1^) that is not considered here^62^. These diffuse CO_2_ sources will also be considered in future developments to the GCP-GridFED protocol.

#### CO_2_ emissions uncertainty

We provide gridded uncertainties to complement all gridded layers of the GCP-GridFED dataset, however we note here the incomplete nature of our uncertainty assessment. The gridded uncertainties are based on the total fossil CO_2_ emissions uncertainty assessment from the GCB^3^, combined with variation in relative uncertainties across emission sectors from the recent TNO assessment (Table 2)^34^ or uncertainties in national total CO_2_ emissions, we adopt the values presented in the uncertainty assessment of the GCB; 5% for the 42 Annex I countries that report annually to the UNFCCC^44^ and 10% for other countries^3,63^ (1σ). Annex I countries are assigned lower uncertainty because for these countries more detailed energy and activity statistics are available, and they are periodically reviewed externally^3^. We used data presented in the TNO uncertainty assessment to evaluate the ratio of the uncertainties for each sector (*U_TNO*_*s*_) to the uncertainty in total emissions (*U_TNO*_*Tot*_). We then scaled the ratios to the uncertainties in total emissions that are adopted for Annex I and other countries from GCB-NAE.

13 $${U}_{GridFEDs}=\left\{\frac{{U}_{TNOs}}{{U}_{TNOTot}}\times 5{\rm{ \% }},\,UNFCCC\,Annex\,I\,\frac{U\_TN{O}_{s}}{U\_TN{O}_{Tot}}\times 10{\rm{ \% }},\,other\right.$$

Table 2 shows sectoral TNO uncertainty estimates and the resulting uncertainties adopted in GCP-GridFED for Annex I and other countries, for each sector.

The gridded uncertainty estimates presented here do not include the uncertainties associated with the spatial or temporal (monthly) disaggregation of national emissions, nor do we present a formal assessment of those disaggregation uncertainties. We note that spatially-averaged uncertainties resulting from the spatial disaggregation of national emissions estimates to grid cells are on the order of 20–75% (1σ) at spatial resolutions of 1 km to 1° (refs. ^53,64–67^). Spatial disaggregation uncertainties occur due to incomplete proxy data coverage (e.g. unmapped or mislocated point sources), poorly constrained nonlinearities (e.g. differences in the emissions intensity between equally dense rural and urban populations), shortcomings in continuous proxy values (e.g. poorly constrained population density) or inappropriate spatial representativeness (e.g. the spatial representativeness of roadmaps for traffic volume). By construction, these uncertainties are larger for years distant from our reference year 2010 and at monthly resolution. Dedicated analyses of regional emissions at high temporal resolution are yielding new data with which to quantify temporal disaggregation uncertainties^57,68^ and to assess the robustness of the temporal profiles employed here and elsewhere^42^.

A full quantitative assessment of these issues, to support the development of comprehensive grid-level uncertainties associated with GCP-GridFED, will be the subject of future work. Overall, our approach to uncertainty quantification is broadly representative of the sectoral contributions to total emissions in each grid cell, which changes throughout the time series. Inversion models may utilise these uncertainty grids but with the freedom to build more complex covariance structures to suit their requirements.

#### O_2_ Uptake

The relationship between CO_2_ and O_2_ fluxes during oxidation reactions can be expressed as an oxidative ratio (OR = flux of O_2_ from the atmosphere/flux of CO_2_ to the atmosphere, unitless)^36,69^. The OR differs detectably between specific fossil fuel sources, holding a value of −1.17 for coal, −1.44 for oil, and −1.95 for natural gas^36,69^. Uncertainties in OR are thought to be on the order of 2–3%, however variations within fuel classes, such as different grades of coal, have not been studied extensively (ref. ^35^). Cement clinker production involves a calcination reaction rather than an oxidation reaction, and thus no exchange of oxygen occurs (OR = 0).

GCP-GridFED calculates gridded estimates of the uptake of O_2_ during fossil fuel oxidation by applying OR values to the CO_2_ emissions estimates for each source.

14 $$GridFED\_O{2}_{S,i,j}=GridFE{D}_{S,i,j}\cdot O{R}_{S}$$

We treat relative uncertainty in O_2_ emissions as equal to the relative uncertainty in CO_2_ emissions (*U_GridFED*).

## Data Records

All GCP-GridFEDv2019.1 output grids can be accessed via the Zenodo data repository^70^ (10.5281/zenodo.3958283).

The data records include 60 files in Network Common Data Form (NetCDF) format with the naming convention GCP_Global_{YYYY}.nc, where YYYY is the year represented by the contents. Each NetCDF file includes 3 dimensions: time (month of the year expressed as days since the first day of YYYY, n = 12); latitude (Degrees North of the equator [cell centres], n = 1800); longitude (Degrees East of the Prime Meridian [cell centres], n = 3600). Each NetCDF file includes three groups representing CO_2_ emissions, CO_2_ emissions uncertainty, and O_2_ uptake (CO2, CO2_uncertainty and O2, respectively). Each group contains five variables representing emissions from each source class (COAL, OIL, GAS, CEMENT, BUNKER) with the units shown in Table 3. Each file contains 1,088,640,000 unique data points. All 60 NetCDF files are contained within a.zip archive named “GCP-GridFEDv2019.1_monthly.zip”.

The data records also include 1 file in NetCDF format, “GCP_Global_Annual.nc”. The NetCDF file includes 3 dimensions: time (year expressed as days since 1959–01–01, n = 60); latitude (Degrees North of the equator [cell centres], n = 1800); longitude (Degrees East of the Prime Meridian [cell centres], n = 3600). Each NetCDF file includes 4 groups representing CO_2_ emissions, CO_2_ emissions uncertainty, and O_2_ uptake, and O_2_ uptake uncertainty (CO_2_, CO_2__uncertainty, O_2_, and O_2__uncertainty respectively). Each group contains 5 variables representing emissions from each source class (COAL, OIL, GAS, CEMENT, BUNKER) with the units shown in Table 4. The file contains 6,998,400,000 unique data points. The NetCDF file is contained within a.zip archive named “GCP-GridFEDv2019.1_annual.zip”.

“GCP-GridFEDv2019.1_monthly.zip” and “GCP-GridFEDv2019.1_annual.zip” can be found within a parent.zip file name “GCP-GridFEDv2019.1.zip”. All grids are bottom-left arranged with coordinates referenced to the prime meridian and the equator.

## Technical Validation

We provide Figs. 2–12, the summary statistics in Tables 4–7 and Online-Only Table 1 to outline the key features of GCP-GridFEDv2019.1 and assist with its technical validation.

GCP-GridFED is designed to distribute national annual emissions from GCB-NAE over a spatio-temporal grid based on EDGARv4.3.2. We validated the outputs from GCP-GridFED by comparing the global annual emissions from the output grids (the sum of emissions across the global grid) with the input data supplied to the gridding protocol from GCB-NAE. Throughout the time series of emissions and across all source classes, the global annual emissions totals from GCP-GridFED were always within 0.0077% of the GCB-NAE input data throughout the annual time series (Figs. 2 and 3). The discrepancies were caused by unscalable (zero or NoData) values in sectors of the EDGAR dataset at the national level in 13 countries (EDGAR data summed within the national masks as per Eq. 10). These 13 countries make a small contribution to total global emissions (0.047% in 2018). Online-Only Table 1 provides national-level comparisons of the emissions estimates from GCP-GridFED and GCB-NAE. For the 13 countries where maximum absolute discrepancies exceeded 1% of GCB-NAE emissions, we provide a brief description of the cause of the discrepancy. The GCP-GridFED outputs are robust to within 0.0001% of GCB-NAE values in 195 countries, plus bunker fuels, comprising 99.9% of global emissions in 2018. Hence, we conclude that the GCP-GridFEDv2019.1 is consistent with GCB-NAE emissions estimates for the years 1959–2018.

We also observed a close match between the seasonality seen in the year 2010 in the GCP-GridFED dataset and that seen in the same year of the EDGAR input data, both at the global scale and in large austral and boreal extratropical nations (Fig. 12). This coherence indicates that the seasonality seen in the EDGAR dataset was preserved by the GCP-GridFED protocol. Inter-annual variability in the monthly distribution of emissions can be seen most prominently in the EU27 + UK. Note that EDGARv4.3.2 does not feature monthly variability in emissions for tropical countries^41^, and so GCP-GridFED also shows no seasonality in these countries.

## Usage Notes

The data is intended for use as a prior in inversion model studies, which may wish to incorporate individual priors for each source class or to use total gridded emissions. The data records contain a layer for each source class. Global total emissions can be calculated as the sum of emissions across the 5 source classes. National total emissions estimates should be calculated as the sum of coal, oil, gas and cement emissions (bunker fuel emissions should not be included in national emissions totals)^71^.

GCP-GridFED will be updated annually and made available for the inversion model runs conducted annually as part of the GCP assessment of the GCB. An updated version of GCP-GridFED (GCP-GridFEDv2020.1) was already made available upon request to support the inversion model runs of the GCP’s 2020 GCB assessment^72,73^ and is now publicly available^74^. GCP-GridFEDv2020.1 is based on the emissions estimates from a preliminary release of GCB-NAE covering the years 1959–2019 with input data available to June 2020. Further updates will be issued as the GCB-NAE data is updated^74^.

When using GCP-GridFED as a prior in inversion models operating at a coarser resolution, aggregation to the required resolution should account for the non-independence of gridded emissions uncertainties. See ‘CO_2_ Emissions Uncertainty’ for further information regarding our treatment of spatial and temporal aggregation in GCP-GridFED.

## Data Availability

The code used to perform all steps described here and shown in Fig. 1 can be accessed via the Zenodo dataset repository entry for GCP-GridFEDv2020.1 (10.5281/zenodo.4277267)^74^. GCP-GridFEDv2020.1 uses the same code and methodology as GCP-GridFEDv2019.1 but includes updated estimates of national annual emissions through to 2019 from the GCP, as discussed in the Usage Notes and also detailed at ref. ^74^.

## Online-only Table

**Online-Only Table 1:** Statistical summary of the differences between national annual emissions estimates in the output GridFEDv2019.1 relative to the input GCB-NAE data in the period 1959–2018.

ISO3 Code	Country	Min	Max	Mean	Explanation for Differences >1%
AFG	Afghanistan	0.00%	0.00%	0.00%	
ALB	Albania	0.00%	0.00%	0.00%	
DZA	Algeria	0.00%	0.00%	0.00%	
AND	Andorra	0.00%	0.00%	0.00%	
AGO	Angola	0.00%	0.00%	0.00%	
AIA	Anguilla	0.00%	0.00%	0.00%	
ATG	Antigua and Barbuda	0.00%	0.00%	0.00%	
ARG	Argentina	0.00%	0.00%	0.00%	
ARM	Armenia	0.00%	0.00%	0.00%	
ABW	Aruba	0.00%	0.00%	0.00%	
AUS	Australia	0.00%	0.00%	0.00%	
AUT	Austria	0.00%	0.00%	0.00%	
AZE	Azerbaijan	0.00%	0.00%	0.00%	
CIV	Côte d’Ivoire	0.00%	0.00%	0.00%	
BHS	Bahamas	0.00%	0.00%	0.00%	
BHR	Bahrain	0.00%	0.00%	0.00%	
BGD	Bangladesh	0.00%	0.00%	0.00%	
BRB	Barbados	0.00%	0.00%	0.00%	
BLR	Belarus	0.00%	0.00%	0.00%	
BEL	Belgium	0.00%	0.00%	0.00%	
BLZ	Belize	0.00%	0.00%	0.00%	
BEN	Benin	0.00%	0.00%	0.00%	
BMU	Bermuda	−0.80%	0.00%	−0.01%	
BTN	Bhutan	0.00%	0.00%	0.00%	
BOL	Bolivia	0.00%	0.00%	0.00%	
BES	Bonaire, Saint Eustatius and Saba	0.00%	0.00%	0.00%	
BIH	Bosnia and Herzegovina	0.00%	0.00%	0.00%	
BWA	Botswana	0.00%	0.00%	0.00%	
BRA	Brazil	0.00%	0.00%	0.00%	
VGB	British Virgin Islands	0.00%	0.00%	0.00%	
BRN	Brunei Darussalam	−0.05%	0.00%	0.00%	
BGR	Bulgaria	0.00%	0.00%	0.00%	
BFA	Burkina Faso	0.00%	0.00%	0.00%	
BDI	Burundi	0.00%	0.00%	0.00%	
KHM	Cambodia	0.00%	0.00%	0.00%	
CMR	Cameroon	0.00%	0.00%	0.00%	
CAN	Canada	0.00%	0.00%	0.00%	
CPV	Cape Verde	0.00%	0.00%	0.00%	
CAF	Central African Republic	0.00%	0.00%	0.00%	
TCD	Chad	0.00%	0.00%	0.00%	
CHL	Chile	0.00%	0.00%	0.00%	
CHN	China	0.00%	0.00%	0.00%	
COL	Colombia	0.00%	0.00%	0.00%	
COM	Comoros	0.00%	0.00%	0.00%	
COG	Congo	0.00%	0.00%	0.00%	
COK	Cook Islands	−100.00%	−100.00%	−100.00%	No emissions data for all sectors in EDGARv4.3.2.
CRI	Costa Rica	0.00%	0.00%	0.00%	
HRV	Croatia	0.00%	0.00%	0.00%	
CUB	Cuba	0.00%	0.00%	0.00%	
CUW	Curaçao	0.00%	0.00%	0.00%	
CYP	Cyprus	−0.04%	0.00%	−0.01%	
CZE	Czech Republic	0.00%	0.00%	0.00%	
COD	Democratic Republic of the Congo	0.00%	0.00%	0.00%	
DNK	Denmark	0.00%	0.00%	0.00%	
DJI	Djibouti	0.00%	0.00%	0.00%	
DMA	Dominica	0.00%	0.00%	0.00%	
DOM	Dominican Republic	0.00%	0.00%	0.00%	
ECU	Ecuador	0.00%	0.00%	0.00%	
EGY	Egypt	0.00%	0.00%	0.00%	
SLV	El Salvador	0.00%	0.00%	0.00%	
GNQ	Equatorial Guinea	0.00%	0.00%	0.00%	
ERI	Eritrea	0.00%	0.00%	0.00%	
EST	Estonia	0.00%	0.00%	0.00%	
ETH	Ethiopia	0.00%	0.00%	0.00%	
FRO	Faeroe Islands	−100.00%	−100.00%	−100.00%	No emissions data for all sectors in EDGARv4.3.2.
FJI	Fiji	0.00%	0.00%	0.00%	
FIN	Finland	0.00%	0.00%	0.00%	
FRA	France	0.00%	0.00%	0.00%	
PYF	French Polynesia	0.00%	0.00%	0.00%	
GAB	Gabon	0.00%	0.00%	0.00%	
GMB	Gambia	0.00%	0.00%	0.00%	
GEO	Georgia	0.00%	0.00%	0.00%	
DEU	Germany	0.00%	0.00%	0.00%	
GHA	Ghana	0.00%	0.00%	0.00%	
GRC	Greece	0.00%	0.00%	0.00%	
GRL	Greenland	−54.10%	0.00%	−7.11%	No emissions data for multiple sectors in EDGAR v4.3.2. Sectors present: 2A1, 2A7, 3A, 3B, 3D.
GRD	Grenada	0.00%	0.00%	0.00%	
GTM	Guatemala	0.00%	0.00%	0.00%	
GIN	Guinea	0.00%	0.00%	0.00%	
GNB	Guinea-Bissau	0.00%	0.00%	0.00%	
GUY	Guyana	0.00%	0.00%	0.00%	
HTI	Haiti	0.00%	0.00%	0.00%	
HND	Honduras	0.00%	0.00%	0.00%	
HKG	Hong Kong	0.00%	0.00%	0.00%	
HUN	Hungary	0.00%	0.00%	0.00%	
ISL	Iceland	−5.30%	0.00%	−2.00%	No emissions data for multiple sectors in EDGAR v4.3.2. Sectors present: 1A1a, 1A2, 1A3a, 1A3b, 1A3d, 1A4, 2A1, 2A3, 2A7, 2B, 2 C, 2 G, 3 A, 3B, 3 C, 3D
IND	India	0.00%	0.00%	0.00%	
IDN	Indonesia	0.00%	0.00%	0.00%	
IRN	Iran	0.00%	0.00%	0.00%	
IRQ	Iraq	0.00%	0.00%	0.00%	
IRL	Ireland	0.00%	0.00%	0.00%	
ISR	Israel	0.00%	0.00%	0.00%	
ITA	Italy	0.00%	0.00%	0.00%	
JAM	Jamaica	0.00%	0.00%	0.00%	
JPN	Japan	0.00%	0.00%	0.00%	
JOR	Jordan	0.00%	0.00%	0.00%	
KAZ	Kazakhstan	0.00%	0.00%	0.00%	
KEN	Kenya	0.00%	0.00%	0.00%	
KIR	Kiribati	0.00%	0.00%	0.00%	
KWT	Kuwait	0.00%	0.00%	0.00%	
KGZ	Kyrgyzstan	0.00%	0.00%	0.00%	
LAO	Laos	0.00%	0.00%	0.00%	
LVA	Latvia	0.00%	0.00%	0.00%	
LBN	Lebanon	0.00%	0.00%	0.00%	
LSO	Lesotho	0.00%	0.00%	0.00%	
LBR	Liberia	0.00%	0.00%	0.00%	
LBY	Libya	0.00%	0.00%	0.00%	
LIE	Liechtenstein	0.00%	0.00%	0.00%	
LTU	Lithuania	0.00%	0.00%	0.00%	
LUX	Luxembourg	0.00%	0.00%	0.00%	
MAC	Macao	0.00%	0.00%	0.00%	
MKD	Macedonia (Republic of)	0.00%	0.00%	0.00%	
MDG	Madagascar	0.00%	0.00%	0.00%	
MWI	Malawi	0.00%	0.00%	0.00%	
MYS	Malaysia	0.00%	0.00%	0.00%	
MDV	Maldives	0.00%	0.00%	0.00%	
MLI	Mali	0.00%	0.00%	0.00%	
MLT	Malta	−41.28%	0.00%	−5.76%	No emissions data for multiple sectors in EDGAR v4.3.2. Sectors present: 1A1a, 1A2, 1A3b, 1A4, 2A1, 2A2, 2A3, 2A4, 2A7, 2B, 2 G, 3 A, 3D
MHL	Marshall Islands	−100.00%	−100.00%	−100.00%	No emissions data for all sectors in EDGARv4.3.2.
MRT	Mauritania	0.00%	0.00%	0.00%	
MUS	Mauritius	0.00%	0.00%	0.00%	
MEX	Mexico	0.00%	0.00%	0.00%	
FSM	Micronesia (Federated States of)	−100.00%	−100.00%	−100.00%	No emissions data for all sectors in EDGARv4.3.2.
MDA	Moldova	0.00%	0.00%	0.00%	
MNG	Mongolia	0.00%	0.00%	0.00%	
MNE	Montenegro	0.00%	0.00%	0.00%	
MSR	Montserrat	0.00%	0.00%	0.00%	
MAR	Morocco	0.00%	0.00%	0.00%	
MOZ	Mozambique	0.00%	0.00%	0.00%	
MMR	Myanmar	0.00%	0.00%	0.00%	
NAM	Namibia	0.00%	0.00%	0.00%	
NRU	Nauru	0.00%	0.00%	0.00%	
NPL	Nepal	0.00%	0.00%	0.00%	
NLD	Netherlands	0.00%	0.00%	0.00%	
NCL	New Caledonia	0.00%	0.00%	0.00%	
NZL	New Zealand	0.00%	0.00%	0.00%	
NIC	Nicaragua	0.00%	0.00%	0.00%	
NER	Niger	0.00%	0.00%	0.00%	
NGA	Nigeria	0.00%	0.00%	0.00%	
NIU	Niue	−100.00%	−100.00%	−100.00%	No emissions data for all sectors in EDGARv4.3.2.
PRK	North Korea	0.00%	0.00%	0.00%	
NOR	Norway	0.00%	0.00%	0.00%	
PSE	Occupied Palestinian Territory	0.00%	0.00%	0.00%	
OMN	Oman	0.00%	0.00%	0.00%	
PAK	Pakistan	0.00%	0.00%	0.00%	
PLW	Palau	−100.00%	−100.00%	−100.00%	No emissions data for all sectors in EDGARv4.3.2.
PAN	Panama	0.00%	0.00%	0.00%	
PNG	Papua New Guinea	0.00%	0.00%	0.00%	
PRY	Paraguay	0.00%	0.00%	0.00%	
PER	Peru	0.00%	0.00%	0.00%	
PHL	Philippines	0.00%	0.00%	0.00%	
POL	Poland	0.00%	0.00%	0.00%	
PRT	Portugal	0.00%	0.00%	0.00%	
QAT	Qatar	0.00%	0.00%	0.00%	
ROU	Romania	0.00%	0.00%	0.00%	
RUS	Russian Federation	0.00%	0.00%	0.00%	
RWA	Rwanda	0.00%	0.00%	0.00%	
SHN	Saint Helena	−100.00%	−100.00%	−100.00%	No emissions data for all sectors in EDGARv4.3.2.
KNA	Saint Kitts and Nevis	0.00%	0.00%	0.00%	
LCA	Saint Lucia	0.00%	0.00%	0.00%	
SPM	Saint Pierre and Miquelon	−80.00%	0.00%	−11.05%	No emissions data for multiple sectors in EDGAR v4.3.2. Sectors present: 1A3b, 1A4, 2A1, 2A7.
VCT	Saint Vincent and the Grenadines	0.00%	0.00%	0.00%	
WSM	Samoa	0.00%	0.00%	0.00%	
STP	Sao Tome and Principe	0.00%	0.00%	0.00%	
SAU	Saudi Arabia	0.00%	0.00%	0.00%	
SEN	Senegal	0.00%	0.00%	0.00%	
SRB	Serbia	0.00%	0.00%	0.00%	
SYC	Seychelles	0.00%	0.00%	0.00%	
SLE	Sierra Leone	0.00%	0.00%	0.00%	
SGP	Singapore	0.00%	0.00%	0.00%	
SVK	Slovakia	0.00%	0.00%	0.00%	
SVN	Slovenia	0.00%	0.00%	0.00%	
SLB	Solomon Islands	0.00%	0.00%	0.00%	
SOM	Somalia	0.00%	0.00%	0.00%	
ZAF	South Africa	0.00%	0.00%	0.00%	
KOR	South Korea	0.00%	0.00%	0.00%	
SSD	South Sudan	0.00%	0.00%	0.00%	
ESP	Spain	0.00%	0.00%	0.00%	
LKA	Sri Lanka	0.00%	0.00%	0.00%	
SDN	Sudan	0.00%	0.00%	0.00%	
SUR	Suriname	0.00%	0.00%	0.00%	
SWZ	Swaziland	0.00%	0.00%	0.00%	
SWE	Sweden	0.00%	0.00%	0.00%	
CHE	Switzerland	0.00%	0.00%	0.00%	
SYR	Syria	0.00%	0.00%	0.00%	
TJK	Tajikistan	0.00%	0.00%	0.00%	
TZA	Tanzania	0.00%	0.00%	0.00%	
THA	Thailand	0.00%	0.00%	0.00%	
TLS	Timor-Leste	0.00%	0.00%	0.00%	
TGO	Togo	0.00%	0.00%	0.00%	
TON	Tonga	0.00%	0.00%	0.00%	
TTO	Trinidad and Tobago	0.00%	0.00%	0.00%	
TUN	Tunisia	0.00%	0.00%	0.00%	
TUR	Turkey	0.00%	0.00%	0.00%	
TKM	Turkmenistan	0.00%	0.00%	0.00%	
TCA	Turks and Caicos Islands	0.00%	0.00%	0.00%	
TUV	Tuvalu	0.00%	0.00%	0.00%	
UGA	Uganda	0.00%	0.00%	0.00%	
UKR	Ukraine	0.00%	0.00%	0.00%	
ARE	United Arab Emirates	0.00%	0.00%	0.00%	
GBR	United Kingdom	0.00%	0.00%	0.00%	
URY	Uruguay	0.00%	0.00%	0.00%	
USA	USA	0.00%	0.00%	0.00%	
UZB	Uzbekistan	0.00%	0.00%	0.00%	
VUT	Vanuatu	0.00%	0.00%	0.00%	
VEN	Venezuela	0.00%	0.00%	0.00%	
VNM	Vietnam	0.00%	0.00%	0.00%	
WLF	Wallis and Futuna Islands	−100.00%	−100.00%	−100.00%	No emissions data for all sectors in EDGARv4.3.2.
YEM	Yemen	−3.15%	0.00%	−0.40%	No emissions data for multiple sectors in EDGAR v4.3.2. Sectors present: 1A1a, 1A1bc, 1A2, 1A3b, 1A4, 1B2, 2A1, 2A4, 2A7, 2B, 3 A, 3 C, 3D
ZMB	Zambia	0.00%	0.00%	0.00%	
ZWE	Zimbabwe	0.00%	0.00%	0.00%	
	Bunker	0.00%	0.00%	0.00%	
	World	−0.01%	0.00%	0.00%	

## References

[1] Joos F, Spahni R (2008). Rates of change in natural and anthropogenic radiative forcing over the past 20,000 years. Proc. Natl. Acad. Sci..

[2] IPCC. *Climate Change 2014: Synthesis Report. Contribution of Working Groups I, II and III to the Fifth Assessment Report of the Intergovernmental Panel on Climate Change*. (2014).

[3] Friedlingstein P (2019). Global Carbon Budget 2019. Earth Syst. Sci. Data.

[4] Le Quéré C (2019). Drivers of declining CO_2_ emissions in 18 developed economies. Nat. Clim. Chang..

[5] Peters GP (2017). Towards real-time verification of CO_2_ emissions. Nat. Clim. Chang..

[6] Ciais, P. *et al*. Carbon and Other Biogeochemical Cycles. In *Climate Change 2013: The Physical Science Basis. Contribution of Working Group I to the Fifth Assessment Report of the Intergovernmental Panel on Climate Change* (ed. Intergovernmental Panel on Climate Change) 465–570 (Cambridge University Press, 2013).

[7] Denman, K. L. *et al*. Couplings Between Changes in the Climate System and Biogeochemistry. In *Climate Change 2007: The Physical Science Basis. Contribution of Working Group I to the Fourth Assessment Report of the Intergovernmental Panel on Climate Change* (eds. Solomon, S. *et al*.) 499–587 (Cambridge University Press, Cambridge, UK and New York, USA, 2007).

[8] Prentice, I. C. *et al*. The Carbon Cycle and Atmospheric Carbon Dioxide. In *Climate Change 2001: The Scientific Basis. Contribution of Working Group I to the Third Assessment Report of the Intergovernmental Panel on Climate Change* (eds. Houghton, J. T. *et al*.) 183–237 (Cambridge University Press, 2001).

[9] Schimel, D. *et al*. Radiative Forcing of Climate Change. In *Climate Change 1995 The Science of Climate Change. Contribution of Working Group I to the Second Assessment Report of the Intergovernmental Panel on Climate Change* (eds. Houghton, J. T. *et al*.) (Cambridge University Press, 1995).

[10] Watson, R. T., Rodhe, H., Oeschger, H., Siegenthaler, U. & Press, C. U. Greenhouse Gases and Aerosols. In *Climate Change: The IPCC Scientific Assessment. Intergovernmental Panel on Climate Change (IPCC)* (eds. Houghton, J. T., Jenkins, G. J. & Ephraums, J. J.) 1–40 (Cambridge University Press, 1990).

[11] Le Quéré C (2013). The global carbon budget 1959–2011. Earth Syst. Sci. Data.

[12] Canadell JG (2007). Contributions to accelerating atmospheric CO_2_ growth from economic activity, carbon intensity, and efficiency of natural sinks. Proc. Natl. Acad. Sci..

[13] Friedlingstein P (2010). Update on CO_2_ emissions. Nat. Geosci..

[14] BP. *Statistical Review of World Energy, 2019*. https://www.bp.com/content/dam/bp/business-sites/en/global/corporate/pdfs/energy-economics/statistical-review/bp-stats-review-2019-full-report.pdf (2019).

[15] Gilfillan, D., Marland, G., Boden, T. & Andres, R. *Global, Regional, and National Fossil-Fuel CO*_*2*_*Emissions*. https://energy.appstate.edu/CDIAC (2019).

[16] Andrew RM (2019). Global CO_2_ emissions from cement production, 1928–2018. Earth Syst. Sci. Data.

[17] Hansis E, Davis SJ, Pongratz J (2015). Relevance of methodological choices for accounting of land use change carbon fluxes. Global Biogeochem. Cycles.

[18] Houghton RA, Nassikas AA (2017). Global and regional fluxes of carbon from land use and land cover change 1850–2015. Global Biogeochem. Cycles.

[19] Sitch S (2015). Recent trends and drivers of regional sources and sinks of carbon dioxide. Biogeosciences.

[20] Hauck J (2020). Consistency and Challenges in the Ocean Carbon Sink Estimate for the Global Carbon Budget. Front. Mar. Sci..

[21] Stephens BB (2007). Weak northern and strong tropical land carbon uptake from vertical profiles of atmospheric CO_2_. Science (80-.)..

[22] Gurney KR (2002). Towards robust regional estimates of annual mean {CO}_2 sources and sinks. Nature.

[23] Rödenbeck C, Zaehle S, Keeling R, Heimann M (2018). How does the terrestrial carbon exchange respond to inter-annual climatic variations? A quantification based on atmospheric CO_2_ data. Biogeosciences.

[24] Van Der Laan-Luijkx IT (2017). The CarbonTracker Data Assimilation Shell (CTDAS) v1.0: Implementation and global carbon balance 2001-2015. Geosci. Model Dev..

[25] Chevallier F (2005). Inferring CO 2 sources and sinks from satellite observations: Method and application to TOVS data. J. Geophys. Res..

[26] Peters W (2010). Seven years of recent European net terrestrial carbon dioxide exchange constrained by atmospheric observations. Glob. Chang. Biol..

[27] Basu S, Miller JB, Lehman S (2016). Separation of biospheric and fossil fuel fluxes of CO_2_ by atmospheric inversion of CO_2_ and 14CO_2_ measurements: Observation System Simulations. Atmos. Chem. Phys..

[28] Saeki T, Patra PK (2017). Implications of overestimated anthropogenic CO_2_ emissions on East Asian and global land CO_2_ flux inversion. Geosci. Lett..

[29] Gaubert B (2019). Global atmospheric CO_2_ inverse models converging on neutral tropical land exchange, but disagreeing on fossil fuel and atmospheric growth rate. Biogeosciences.

[30] Rödenbeck C, Houweling S, Gloor M, Heimann M (2003). CO_2_ flux history 1982–2001 inferred from atmospheric data using a global inversion of atmospheric transport. Atmos. Chem. Phys..

[31] Chevallier F (2010). CO_2_ surface fluxes at grid point scale estimated from a global 21 year reanalysis of atmospheric measurements. J. Geophys. Res..

[32] Peters W (2005). An ensemble data assimilation system to estimate CO 2 surface fluxes from atmospheric trace gas observations. J. Geophys. Res..

[33] Friedlingstein, P. *et al*. Supplemental data of the Global Carbon Budget 2019. *ICOS-ERIC Carbon Portal*10.18160/gcp-2019 (2019).

[34] Marshall, J. & Ramirez, T. *CHE Project Deliverable 4.3 Attribution Problem Configurations*. https://www.che-project.eu/sites/default/files/2020-01/CHE-D4-3-V4-1.pdf (2019).

[35] Keeling, R. F. & Manning, A. C. Studies of Recent Changes in Atmospheric O_2_ Content. In *Treatise on Geochemistry* (eds. Holland, H. D. & Turekian, K. K.) **5**, 385–404 (2014).

[36] Steinbach J (2011). The CO_2_ release and Oxygen uptake from Fossil Fuel Emission Estimate (COFFEE) dataset: Effects from varying oxidative ratios. Atmos. Chem. Phys..

[37] Gruber N, Gloor M, Fan SM, Sarmiento JL (2001). Air-sea flux of oxygen estimated from bulk data: Implications for the marine and atmospheric oxygen cycles. Global Biogeochem. Cycles.

[38] Tohjima Y, Mukai H, Nojiri Y, Yamagishi H, Machida T (2008). Atmospheric O2/N2 measurements at two Japanese sites: Estimation of global oceanic and land biotic carbon sinks and analysis of the variations in atmospheric potential oxygen (APO). Tellus, Ser. B Chem. Phys. Meteorol..

[39] Rödenbeck C, Le Quéré C, Heimann M, Keeling RF (2008). Interannual variability in oceanic biogeochemical processes inferred by inversion of atmospheric O2/N2 and CO_2_ data. Tellus, Ser. B Chem. Phys. Meteorol..

[40] Goto D, Morimoto S, Aoki S, Patra PK, Nakazawa T (2017). Seasonal and short-term variations in atmospheric potential oxygen at Ny-Ålesund, Svalbard. Tellus, Ser. B Chem. Phys. Meteorol..

[41] Janssens-Maenhout G (2019). EDGAR v4.3.2 Global Atlas of the three major greenhouse gas emissions for the period 1970–2012. Earth Syst. Sci. Data.

[42] Crippa M (2020). High resolution temporal profiles in the Emissions Database for Global Atmospheric Research. Sci. Data.

[43] Keeling CD (1976). Atmospheric Carbon-Dioxide Variations at Mauna-Loa Observatory, Hawaii. Tellus.

[44] UNFCCC. *National Inventory Submissions*. https://unfccc.int/process-and-meetings/transparency-and-reporting/reporting-and-review-under-the-convention/greenhouse-gas-inventories-annex-i-parties/national-inventory-submissions-2019. (2019).

[45] UNSD. *United Nations Statistics Division: Energy Statistics*. http://unstats.un.org/unsd/energy/. (2019).

[46] Rypdal, K. & Paciornik, N. *2006 IPCC Guidelines for National Greenhouse Gas Inventories, Volume 1: General Guidance and Reporting, Prepared by the National Greenhouse Gas Inventories Programme*. (Intergovernmental Panel on Climate Change, 2006).

[47] IPCC. *Revised 1996 IPCC Guidelines for National Greenhouse Gas Inventories IPCC/OECD/ IEA*. (1996).

[48] Janssens-Maenhout, G., Pagliari, V., Guizzardi, D. & Muntean, M. *Global emission inventories in the Emission Database for Global Atmospheric Research (EDGAR) – Manual (I): Gridding: EDGAR emissions distribution on global grid-maps, JRC Report, EUR 25785 EN*. https://publications.jrc.ec.europa.eu/repository (2013).

[49] European Commission Eurostat (ESTAT) GISCO. *Countries, 2016 - Administrative Units - Dataset ID 5C27B6C0-BC1C-4175-9B0B-783AEEBAAD61*. http://ec.europa.eu/eurostat/web/gisco/geodata/reference-data/administrative-units-statistical-units (2018).

[50] Peters GP (2020). Carbon dioxide emissions continue to grow amidst slowly emerging climate policies. Nat. Clim. Chang..

[51] Ummel, K. Carma Revisited: An Updated Database of Carbon Dioxide Emissions from Power Plants Worldwide. *SSRN Electron. J*. 10.2139/ssrn.2226505 (2012).

[52] Oda T, Maksyutov S, Andres RJ (2018). The Open-source Data Inventory for Anthropogenic CO_2_, version 2016 (ODIAC2016): A global monthly fossil fuel CO_2_ gridded emissions data product for tracer transport simulations and surface flux inversions. Earth Syst. Sci. Data.

[53] Andres RJ, Boden TA, Higdon DM (2016). Gridded uncertainty in fossil fuel carbon dioxide emission maps, a CDIAC example. Atmos. Chem. Phys..

[54] Harris, I., Osborn, T. J., Jones, P. & Lister, D. Version 4 of the CRU TS monthly high-resolution gridded multivariate climate dataset. *Sci. Data* **7**, 109, 10.1038/s41597-020-0453-3 (2020). 10.1038/s41597-020-0453-3 PMC7125108 32246091

[55] Spinoni J (2018). Changes of heating and cooling degree-days in Europe from 1981 to 2100. Int. J. Climatol..

[56] Spinoni J, Vogt J, Barbosa P (2015). European degree-day climatologies and trends for the period 1951-2011. Int. J. Climatol..

[57] Andrew RM (2020). Timely estimates of India’s annual and monthly fossil CO_2_ emissions. Earth Syst. Sci. Data.

[58] EIA. *Short-Term Energy Outlook*. http://www.eia.gov/forecasts/steo/outlook.cfm (2019).

[59] Olivier, J. G. J., Janssens-Maenhout, G., Muntean, M. & Peters, J. A. H.. *Trends in global CO*_*2*_*emissions: 2016 report, JRC 103425*. http://edgar.jrc.ec.europa.eu/overview.php?v=CO2andGHG1970-2016 (2016).

[60] Janssens-Maenhout, G. *et al*. *EDGARv4.3.2_CO*_*2*_*_FT2015 gridmaps*. https://edgar.jrc.ec.europa.eu/overview.php?v=che_h2020. (2019).

[61] Zheng B (2019). Global atmospheric carbon monoxide budget 2000–2017 inferred from multi-species atmospheric inversions. Earth Syst. Sci. Data.

[62] Saunois M (2020). The Global Methane Budget 2000–2017. Earth Syst. Sci. Data.

[63] Andres RJ (2012). A synthesis of carbon dioxide emissions from fossil-fuel combustion. Biogeosciences.

[64] Hogue S, Marland E, Andres RJ, Marland G, Woodard D (2016). Uncertainty in gridded CO_2_ emissions estimates Earth’ s Future. Earth’s Fu.

[65] Super I, Dellaert SNC, Visschedijk AJH, Van Der Gon HACD (2020). Uncertainty analysis of a European high-resolution emission inventory of CO_2_ and CO to support inverse modelling and network design. Atmos. Chem. Phys..

[66] Oda T (2019). Errors and uncertainties in a gridded carbon dioxide emissions inventory. Mitig. Adapt. Strateg. Glob. Chang..

[67] Han P (2020). Evaluating China’s fossil-fuel CO_2_ emissions from a comprehensive dataset of nine inventories. Atmos. Chem. Phys..

[68] EIA. *U.S. Energy Information Administration, Short-Term Energy Outlook*, http://www.eia.gov/forecasts/steo/outlook.cfm (2020).

[69] Keeling, R. F. *Development of an interferometric analyzer for precise measurements of the atmospheric oxygen mole fraction*. (Harvard University, 1988).

[70] Jones MW (2020). Zenodo.

[71] Waldron, C. D., *et al*. 2006 IPCC Guidelines for National Greenhouse Gas Inventories, Volume 2: Energy in *2006 IPCC Guidelines for National Greenhouse Gas Inventories* (ed. Eggleston, S., *et al*.) (Intergovernmental Panel on Climate Change, 2006).

[72] Friedlingstein, P. *et al*. Global Carbon Budget 2020. *Earth Syst. Sci. Data* **12**, 3269–3340, 10.5194/essd-12-3269-2020 (2020).

[73] Friedlingstein, P. *et al*. Supplemental data of the Global Carbon Budget 2020, available at: 10.18160/gcp-2020, ICOS-ERIC Carbon Portal, last accessed: 16 November 2020. 10.18160/gcp-2020 (2020).

[74] Jones MW (2020). Zenodo.

